# The development of an electrochemical immunosensor utilizing chicken IgY anti-spike antibody for the detection of SARS-CoV-2

**DOI:** 10.1038/s41598-023-50501-w

**Published:** 2024-01-07

**Authors:** Khaled M. Al-Qaoud, Yusra M. Obeidat, Tareq Al-Omari, Mohammad Okour, Mariam M. Al-Omari, Mohammad I. Ahmad, Raed Alshadfan, AbdelMonem M. Rawashdeh

**Affiliations:** 1 Department of Biological Sciences, Faculty of Science, Yarmouk University, Irbid, Jordan; 2 Department of Electronics Engineering, Hijjawi Faculty for Engineering Technology, Yarmouk University, Irbid, Jordan; 3 Department of Basic Medical Sciences, Faculty of Medicine, Yarmouk University, Irbid, Jordan; 4 Rawgene Biotech, Umm Khelad St. 33, Amman, Jordan; 5 Atlas Medical, Sahab Industrial Area, Amman, Jordan; 6 Department of Chemical Sciences, Faculty of Pharmacy, University of Porto, Porto, Portugal; 7 Department of Chemistry, Faculty of Science, Yarmouk University, Irbid, Jordan

**Keywords:** Biological techniques, Biotechnology, Diseases

## Abstract

This paper introduces a novel approach for detecting the SARS-CoV-2 recombinant spike protein combining a label free electrochemical impedimetric immunosensor with the use of purified chicken IgY antibodies. The sensor employs three electrodes and is functionalized with an anti-S IgY antibody, ELISA and immunoblot assays confirmed the positive response of chicken immunized with SARS-CoV2 S antigen. The developed immunosensor is effective in detecting SARS-CoV-2 in nasopharyngeal clinical samples from suspected cases. The key advantage of this biosensor is its remarkable sensitivity, and its capability of detecting very low concentrations of the target analyte, with a detection limit of 5.65 pg/mL. This attribute makes it highly suitable for practical point-of-care (POC) applications, particularly in low analyte count clinical scenarios, without requiring amplification. Furthermore, the biosensor has a wide dynamic range of detection, spanning from 11.56 to 740 ng/mL, which makes it applicable for sample analysis in a typical clinical setting.

## Introduction

Solid-support interfaces play a crucial role in biorecognition reactions involving various analytes such as cells, microbes, DNA, proteins, enzymes, antibodies, and antigens^1–3^. These reactions cause significant changes at three key components: the immobilized macromolecule, the macromolecule-fluid interface within the fluid boundary layer, and the bulk fluid surrounding the macromolecule. These changes impact optical and electric properties, including refractive index, voltage, current, capacitance, and impedance. Understanding interfacial interactions is vital for applications like surface wetting, cell adhesion, and biosensor technologies in diverse biological systems^4^.

Compared to optical, colorimetric, and piezoelectric biosensors, electrochemical biosensors are gaining popularity due to their affordability, portability, simplicity, rapidity, sensitivity, and suitability for point-of-care diagnostics, particularly in resource-limited regions^5–10^. Electrochemical impedance spectroscopy (EIS) is widely used in corrosion monitoring, electrodeposition, batteries, biotechnology, drug discovery, disease detection, tissue engineering, cell culture, and environmental monitoring^6–11^. EIS biosensors can also be used in clinical applications and detection of various analyte^11–15^.

The severe acute respiratory syndrome coronavirus 2 (SARS-CoV-2) has resulted in over five million global deaths^16–18^. SARS-CoV-2 is measured by diagnostic methods such as RT-PCR and immunosensors (ELISA assays and agglutination tests), in addition to other less common tools, including loop-mediated isothermal amplification (LAMP), clustered regularly interspaced short palindromic repeats (CRISPR), plasmonic metasensors, and electrochemical impedance spectroscopy (EIS)^7,8,19–22^.

Efforts have been made to enhance biosensors for SARS-CoV-2 detection, focusing on reproducibility, cost reduction, and achieving optimal detection limits utilized the carboxymethyl chitosan conjugation EDC/NHS method and conducted an electrochemistry spectroscopy experiment on spike protein and its antibody. The immunosensors' specificity has been observed by comparing control samples with varying concentrations of inactivated SARS-CoV-2, demonstrating low detection limits of 0.179 fg/mL and selectivity for proteins other than spike protein^7,8^.

In a study by Mojsoska et al.^23^, a graphene electrode was utilized in an EDC conjugation approach and successfully detected signals above 20 µg/mL within 45 min. Another investigation conducted by Eissa et al.^24^ achieved a detection limit of 0.8 pg/mL for N protein in 30 min. Similarly, in 2020, researchers employed similar methods and reported detection limits of 10 fM within 30 s for saliva and 1 fg/mL for nasopharyngeal samples^25,26^.

In this work, we have developed an electrochemical biosensor based on impedance spectroscopy and cyclic voltammetry to detect SARS-CoV-19 spike protein. The immunosensor, equipped with three electrodes and functionalized with anti-S IgY antibodies obtained from purified Polyclonal Chicken IgY antibodies which demonstrates high specificity and sensitivity. The process involved in immobilizing the Anti-Spike protein IgY antibody onto the electrodes included chemisorbing the thiol sites of cysteamine onto the surface of gold nanoparticles. Subsequently, glutaraldehyde was applied to establish a connection between the cysteamine and glutaraldehyde groups, ensuring the linkage of the antibody's amine groups to the aldehyde groups of glutaraldehyde. The developed immunosensor effectively detects SARS-CoV-2 in nasopharyngeal clinical samples from suspected cases. Its remarkable sensitivity allows it to detect very low concentrations of the target analyte, with a detection limit of 5.65 pg/mL. This high sensitivity makes it suitable for practical point-of-care (POC) applications, especially in low target count clinical scenarios, without the need for amplification. Additionally, the biosensor has an extensive dynamic range of detection, ranging from 11.56 to 740 ng/µL, making it applicable for sample analysis in a typical clinical setting.

## Materials and methods

In this section, we will describe the methods and materials used in this work.

### Chicken accommodation for experiment

Animal Experiments were approved by Yarmouk University Animal Care and Use Committee (No.AICUC/2021/11) following Jordanian and US National Institute of health guidelines. All studies involving animals. The study in this work is reported in accordance with ARRIVE guidelines.

Two rooms in the animal house at Yarmouk University were reconstructed for the accommodation of the 22 weeks old Novogen White (Longhorn) egg layer hens. After area disinfection, straw was spread on floor and clean feeder, drinks and buckets were used. Light was programmed in such a way it is on for 18 h and off for 6 h, and rooms temperature was maintained at 26 °C. Healthy hens were purchased from a local farm. Eggs laying was observed for each hen and collected daily and kept in a refrigerator until used.

### Chicken immunization and production of anti-S IgY

Chicken (3 per group) were immunized intramuscularly in the chest with pure antigens of SARS-CoV-2 proteins (S) containing 400 µg mixed 1:1 with complete or incomplete Freund’s adjuvant (CFA) (Thermo Fisher, USA) following a standard immunization protocol. First injection was prepared with FCA and three shots with FIA were given 2 weeks a part. Control group was injected with normal saline mixed with matching adjuvant. Blood samples were collected from immunized and control chicken in plain vacutainer tubes (FL Medical, Italy) and left to clot. Serum was collected after centrifugation of tubes at 2000×*g* (MPW-251, Poland) for 10 min.

### Evaluation of anti S IgY by ELISA

Antibodies binding activities were tested by ELISA. In brief, 96-well plate (Greiner Bio-One, Germany) was coated with 5 µg/mL recombinant S antigens (RayBiotech, USA) prepared in carbonate-bicarbonate buffer (0.05 M, pH 9.6) (PS Park, UK). Purified anti- antibodies with different dilutions were used to determine the antibodies titer. Finally, HRP-conjugated Rabbit anti-IgY (abcam, UK) diluted 1:1000 was added as tracer. Optical density was measured after the addition of substrate solution (TMB, Sigma-Aldrich, Germany) at 450 nm using Multiskan™ GO Microplate Spectrophotometer (Thermo Fisher, USA).

### Extraction of chicken IgY from eggs yolk

Chicken IgY was prepared using the PEG technique. Briefly, egg white-free yolk was collected in 50 mL tubes and mixed with twice volume PBS (Vivantis, Malaysia) and 3.5% poly ethylene glycol (PEG) 6000 (Bio basic, Canada) then vortexed by vortex (Velp Scientifica, Italy), followed by 30 min rolling on a rolling mixer (BIOBASE, China). Aqueous phase was transferred to a new tube and 9.5% PEG 6000 was added then vortexed and rolled for 15 min. Centrifugation step was repeated and pellet that contains precipitated IgY was collected and dissolved again in 12% PEG 6000 and treated as before. After centrifugation the pellet is dissolved in PBS and dialyzed overnight with 1 L of PBS using cellulose membrane (Sigma-Aldrich, Germany). The protein content (mg/mL) of the dialyzed samples was measured by the Bradford protein assay (Sigma-Aldrich, USA) and samples were stored at -20C until further use.

### Purification of polyclonal chicken antibodies using HiTrap IgY column

HiTrap IgY purification column (Cytiva, Sweden), is a prepacked, ready to use, column for purification of IgY from egg yolk. In brief, the column was loaded with 5 mL crude egg PEG extract diluted in binding buffer after being washed with 5 × binding buffer (20 mM sodium phosphate, 0.5 M K_2_SO_4_, pH 7.5). The column was washed with 10 × binding buffer until no material appears in the effluent. Chicken IgY was eluted using elution buffer (20 mM sodium phosphate, pH 7.5). The eluent was concentrated by Vivispin concentrator with MWCO of 30 kDa (Merck Millipore, USA). Antibodies were washed twice with PBS, pH 7.4 and stored at − 20 °C for further processing.

### Characterization of purified chicken IgY using SDS-PAGE

SDS-PAGE was performed for the evaluation of IgY purity. (19:1) Acrylamide/Bisacrylamide, 40% solution (Fisher Biotech, USA) was used. This was performed using Desaphor VE minigel (Cleaver scientific, UK) in the discontinuous buffer system using 0.5 mm thick 12% acrylamide–bisacrylamide gels under reducing conditions. Ten microliter of egg extract or purified IgY were mixed with an equal volume of sample buffer (pH 6.8) and loaded to gel after being boiled for 5 min. For band size determination, molecular weight prestained protein standard (Bio-helix, New Taipei City, Taiwan), was used after being processed in a similar way as extract samples. Electrophoresis was carried out using running buffer with pH 8.3 at 120 V for 60–120 min. The gel was stained with Coomassie brilliant blue R-250 (BDH pool, Dorset, UK), and destained by 20% acetic acid (LOBA Chemie, India) until clear bands were seen.

### Identification of antibody-protein reactivity by western blot

The reactivity of IgY anti S antigen was determined by western blot**.** Recombinant SARS-CoV2 antigens were electrophoresed in 12% SDS-PAGE gel, transferred into the nitrocellulose membrane, and blocked with 3% bovine serum albumin. The membrane was incubated with purified chicken IgY and incubated for 1 h at RT. On a plate rocker. After membrane washing, monoclonal mouse anti-chicken IgY (H&L) HRP conjugated (MY BioSource, USA) diluted 1:1000 in PBS-Tween was added and incubated for 1 h at RT. Ready to use TMB substrate was poured on washed strips until color development.

### Sensor fabrication

**Figure 1:**
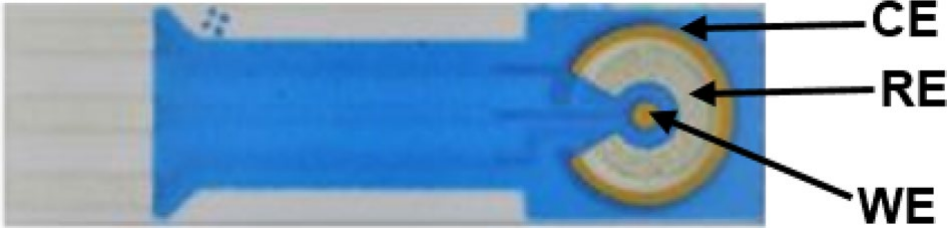
Gold sensors (BVT technologies, AC1W1R1) showing counter electrode (silver), reference electrode (carbon) and working electrode (gold).

In all experiments, a three electrodes gold sensor (manufactured by BVT technologies, Czech Republic) was consistently employed. It consists of a counter electrode (CE) and a working electrode (WE) both are made of gold, and a silver/silver chloride reference electrode (RE), as illustrated in Fig. 1. The biosensor preparation involved several stages. Initially, the electrode surface was rinsed with deionized water and left to dry at room temperature (RT). After the cleaning process, the fabrication proceeded by adding 3 µL of 2 mM cysteamine (Sigma-Aldrich, Germany) exclusively to the working electrode. This step aimed to chemisorb the thiol sites of cysteamine onto the surface of gold nanoparticles. Subsequently, a coating of 30 µL of 5 mM glutaraldehyde (Alfa Aesar, Germany) was applied to the electrode to create a bond between cysteamine and glutaraldehyde groups. This was followed by the addition of 30 µL of 5 µg anti-spike protein IgY antibody, which linked the antibody’s amine groups to the aldehyde groups of glutaraldehyde. Then 30 µL of 5 mM ethanolamine (Sigma-Aldrich, Germany) was applied to the electrode to prevent unbound spaces. All these preparation steps involved a 10-min incubation at room temperature (23 °C) controlled using the room air conditioner.

### Measurement procedure

To detect the spike protein, the electrodes surface was prepared to create the biosensor before each experiment. The biosensor was coated with 30 µL of SARS-CoV-2 spike (S) protein (740, 370, 185, 92.5, 46.25, 23.125, and 11.56 ng/mL), and incubated for 15 min at RT. Before doing electrochemical measurements, biosensors were cleansed with deionized water, rinsed with 1 mL of PBS, and then dried at room temperature.

In all experiments, cyclic voltammetry (CV) and electrochemical impedance spectroscopy (EIS) were performed using a versatile portable potentiostat (PalmSens4 BV, v5.9, Netherlands). The measurements were carried out using PBS containing 5 mM potassium ferri/ferrocyanide [Fe(CN)_6_]^3−^/[Fe(CN)_6_]^4−^ (Thermo Fisher, USA). All CVs were conducted in a voltage range of − 0.2 to 0.5 V with a potential step of 0.01 V and a scan rate of 0.1 V/s. In the EIS measurements, the frequency range was 100 kHz to 0.5 Hz, and the AC and DC voltages were 0.005 V and 0.0 V, respectively.

To identify the spike protein, the electrochemical signal of the custom sensor is recorded after performing a blocking step using ethanolamine, which serves as the background signal. Subsequently, the sensor is exposed to the analyte for incubation, and the electrochemical signal is measured again. As the concentration of the analyte increases, the results obtained from cyclic voltammetry demonstrate a decrease in the peak current at the activation potential. Additionally, impedance spectroscopy reveals an increase in impedance with the rise in analyte concentration. Finally, to obtain the accurate analyte signal, the background signal is subtracted from each measured analyte signal.

The anti-S immunosensor was then further evaluated to determine the limit of detection (LOD) through dilution ranging from 180 pg/mL down to 2.83 pg/mL of SARS-CoV2 recombinant S antigen. To validate the effectiveness of the anti-spike protein biosensors, random COVID-19-positive nasopharyngeal samples were examined after being pipetted out from lysis solution included in the RAPID assay (ATLAS Biomedical, Jordan) and diluted in PBS to varying dilutions. The samples were collected by a qualified staff in a clinics assigned to do RAPID SARS-CoV-2 antigen test by Ministry of Health in Jordan. Vials containing the sample buffers were the nasopharyngeal swabs were washed were used in this study.

## Results

### Antibody IgY response to SARS-CoV2 recombinant proteins

**Figure 2:**
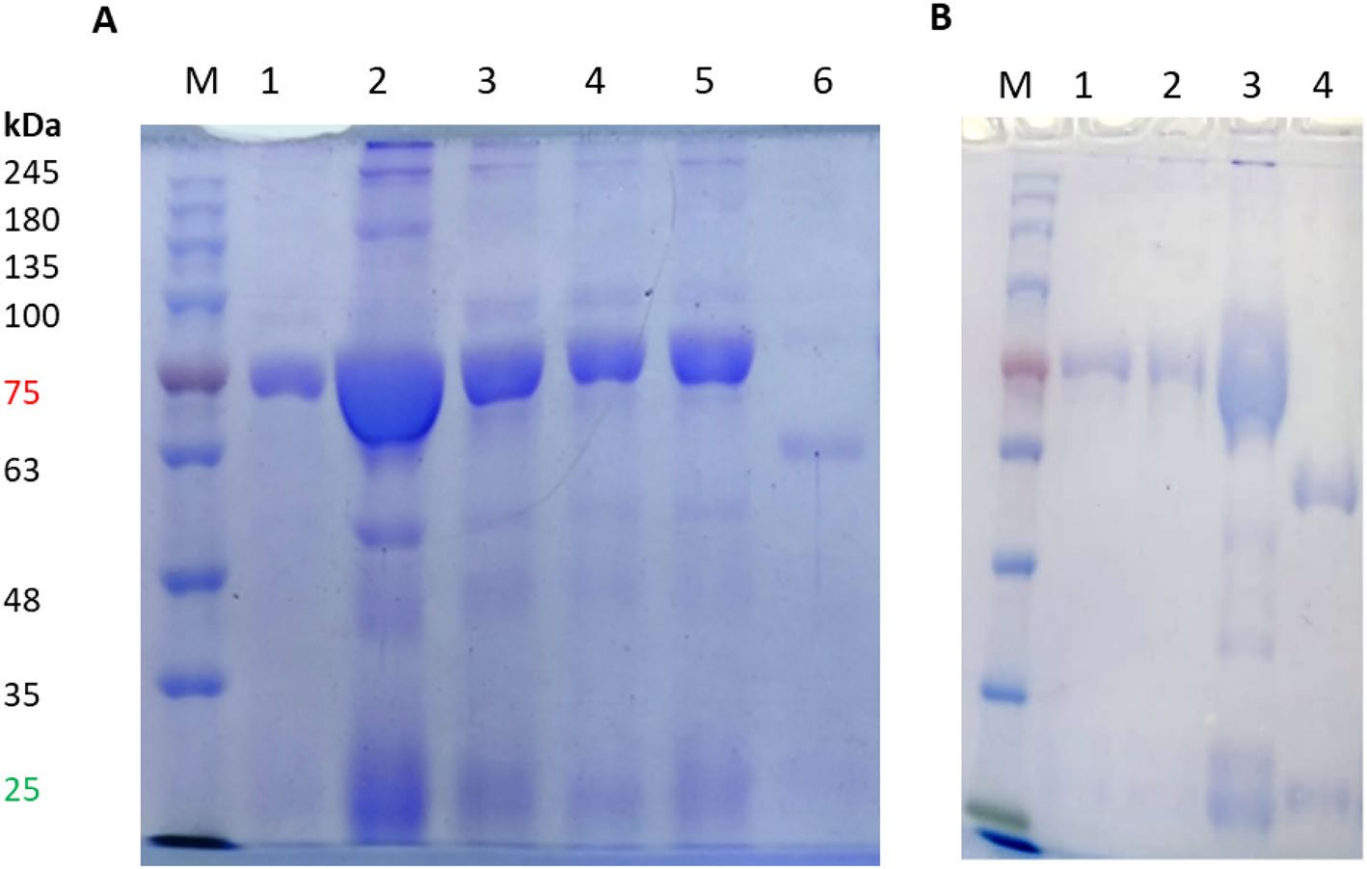
SDS-PAGE of IgY samples collected from chicken immunized with SARS-CoV2 S antigen. M, protein marker, 1A and 1B; anti S IgY purified using HiTrap IgY purification column, 2A; PEG extracted anti S IgY S1, 3A; S2, 4A; S3, 5A; S4, 6A and 4B; affinity purified mouse IgG placed as control.

**Figure 3:**
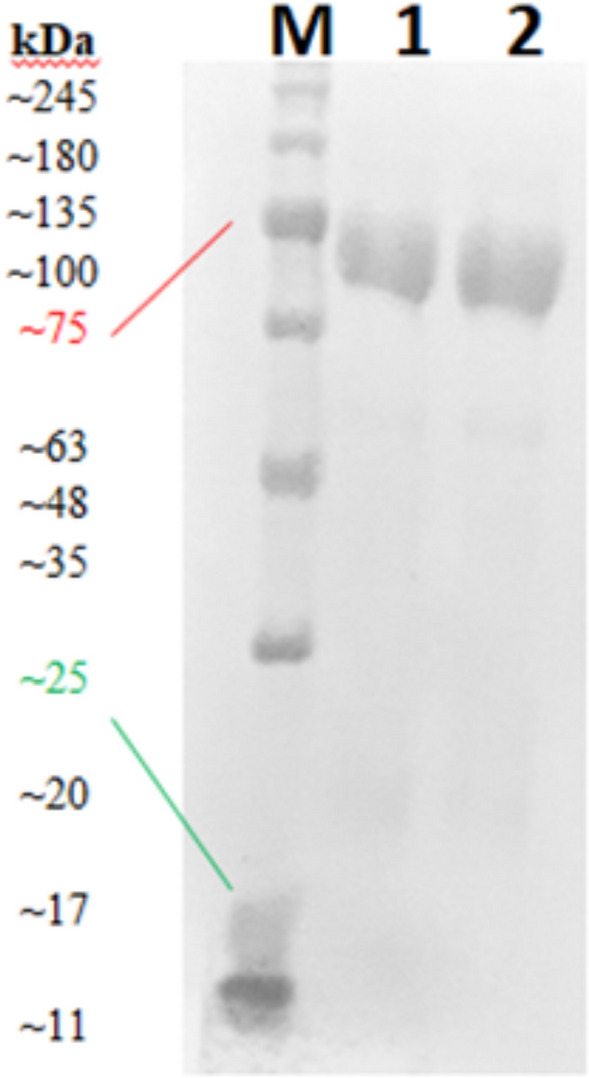
Immunoblot for the reactivity of HiTrap IgY purified IgYs to purified chicken IgY. Commercial HRP conjugated monoclonal mouse anti IgY was used as detection antibody. *M* protein ladder, *1* Purified anti-S IgY Sample 1, *2* purified anti-S IgY sample 2.

Serum samples collected from chicken during the experiment time revealed the production of chicken IgY in response to antigen immunization. ELISA results showed an increase in antibody titer reaching highest concentration after the 3d booster. Chicken laid eggs were collected and IgY was extracted from egg yolk using PEG extraction protocol. The total yield of purified IgY per egg was between 17.5 and 25 mg for about 10 extraction and purification trials. Furthermore, purification of extracted IgY using affinity columns revealed the presence of pure IgY with heavy and light chains of the expected sizes (Fig. 2). The reactivity of the purified anti Covid 19 IgY were confirmed using immunoblot against recombinant antigens (Fig. 3).

### Results from the electrochemical impedance spectroscopy

**Figure 4:**
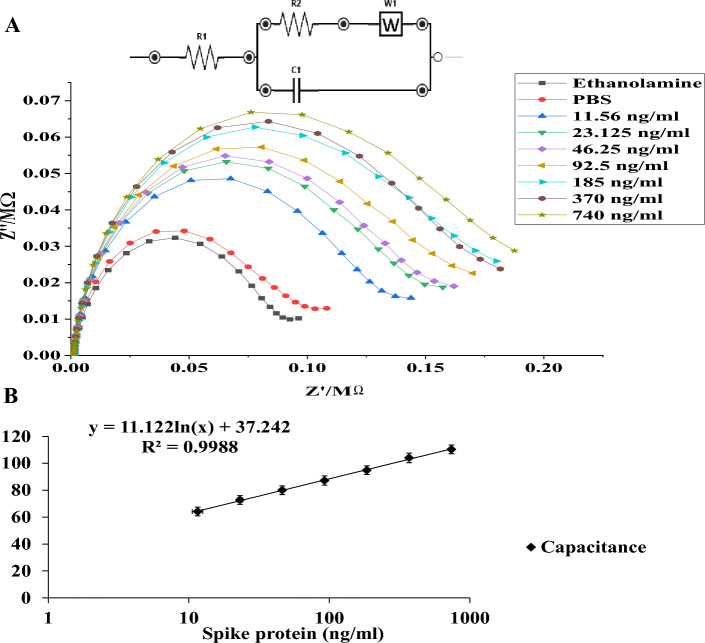
Electrochemical impedance spectroscopy for anti-s protein biosensor; (**A**) Nyquist plot resulted from response to various spike protein concentrations ranging from 11.56 to 740 ng/mL under the effect of ferri/ferrocyanide oxidation–reduction; (**B**) Capacitance curve obtained from several trials (n = 7 for each concentration, mean ± SEM).

The electrochemical impedance spectroscopy (EIS) was employed throughout the experiments to assess the behavior of the electrode after each step of surface modification. The PStrace 5.8 software was used to display the Nyquist Plots that represent the impedance of spike protein at different concentrations ranging from 11.56 to 740 ng/mL (Fig. 4). The subsequent addition of phosphate-buffered saline (PBS) to the sensor as a control analyte resulted in a relatively minor increase in impedance compared to the ethanolamine step. However, introducing spike protein at a concentration of 11.56 ng/mL with a 15-min incubation time significantly increased the measured impedance. Consequently, the measured impedance increased with higher protein concentrations (Fig. 4A). We have included more electrochemical impedance spectroscopy results in the Supplementary file (S.4 Supplementary Figs. 1–6).

A data fitting was done for the measured impedance spectrum and a circuit model was developed to represent a quantitative measurement for the impedance at different protein concentrations. This circuit, depicted in the inset of Fig. 4, consists of different components arranged in a specific configuration: R1, C1, R_2_, and W. It incorporates the solution resistance (Rsol), a double layer capacitance denoted as C1, the charge transfers resistance (R_ct_), and the Warburg impedance (W). The reaction is believed to occur in a single step, and the overall process is described by a combination of kinetic and diffusion processes through a layer that is considered to have infinite thickness. The circuit model, shown in the inset of Fig. 2 presents a parallel arrangement of double layer capacitance C_1_ and a Warburg impedance (W). Additionally, there is a resistance (R_ct_) to account for the accessibility of ions within porous structures and surface functionalities, as well as the polarization resistance. To consider the resistance of the electrolyte and the contact resistance of the current collector, a series resistance (R_sol_) is introduced.

In analytical applications, the Warburg impedance can be disregarded in the complete equivalent circuit by selecting a frequency range where a 45° line is not visible in the Nyquist plot. This is because the Warburg impedance is directly proportional to the square root of the frequency. As a result, at higher frequencies, the contribution from the Warburg impedance becomes negligible due to its small magnitude. Upon analyzing the data and performing fitting procedures, it was observed that the capacitance values in the generated models decreases with the increase in the analyte concentration. By normalizing the capacitance data with the ethanolamine data, the resulting calibration curve exhibited an almost linear rise in capacitance values as the spike protein concentrations increased, ranging from 11.56 to 740 ng/mL (Fig. 4B).

### Results from cyclic voltammetry

**Figure 5:**
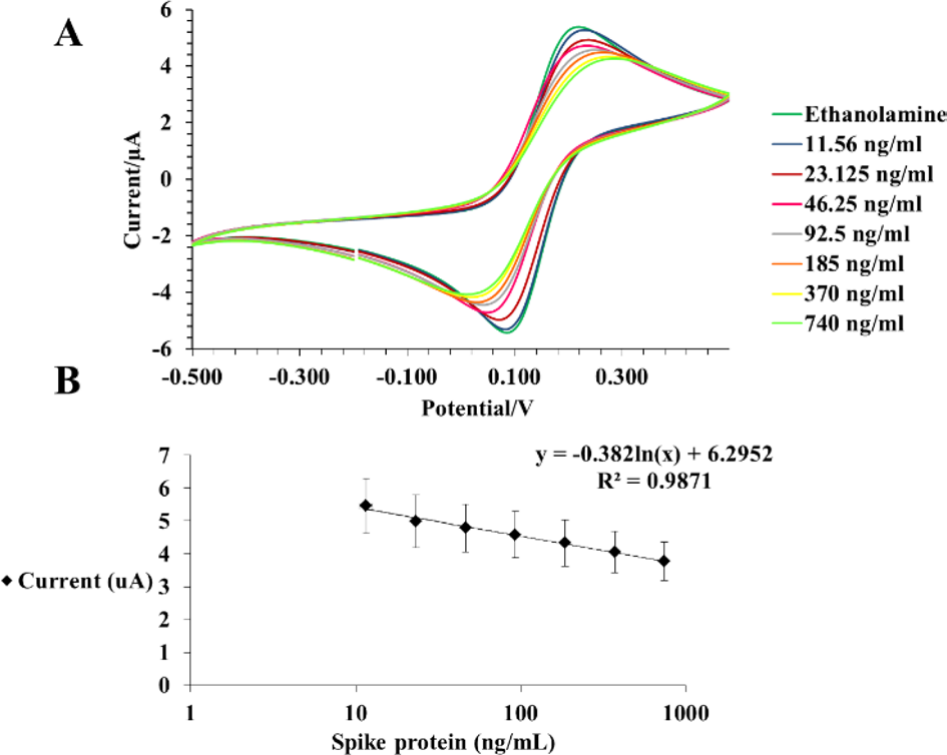
cyclic voltammograms (CVs) of spike protein at different concentrations (ng/mL); (**A**) Cyclic voltammogram of spike protein from 11.56 to 740 ng/mL with activation potential from 0.220 to 0.260 V; (**B**) Cyclic voltammetry of spike protein for several trials (n = 8 for each concentration, mean ± SEM), the activation potential is 0.28 ± 0.35.

The results from cyclic voltammetry confirmed the results achieved from impedance spectroscopy and clearly demonstrated the reversible peaks of the ferri/ferrocyanide electron transfer from one step to another. The results in Fig. 5 show that as the concentration of the analyte increases, the current peak decreases at approximately the same activation voltage. This observation aligns with Ohm's law, which state that current is inversely proportional to impedance. We have included more cyclic voltammetry results in the Supplementary file (S.6 Supplementary Figs. 9–13).

**Figure 6:**
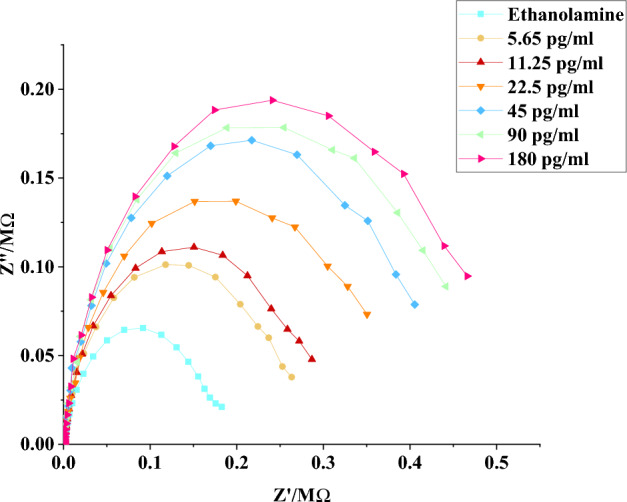
Nyquist plot represent limit of detection (LOD) for spike-protein at pictogram concentrations.

Additionally, the limit of detection (LOD) of the anti-S immunosensor was 5.65 pg/mL (Fig. 6), indicating that the sensor was able to detect very low concentrations of the target analyte, making it a very sensitive and reliable immunosensor. we have included more LOD results in the Supplementary file (S.5 Supplementary Figs. 7, 8).

### Test validation on spike proteins using clinical samples

**Figure 7:**
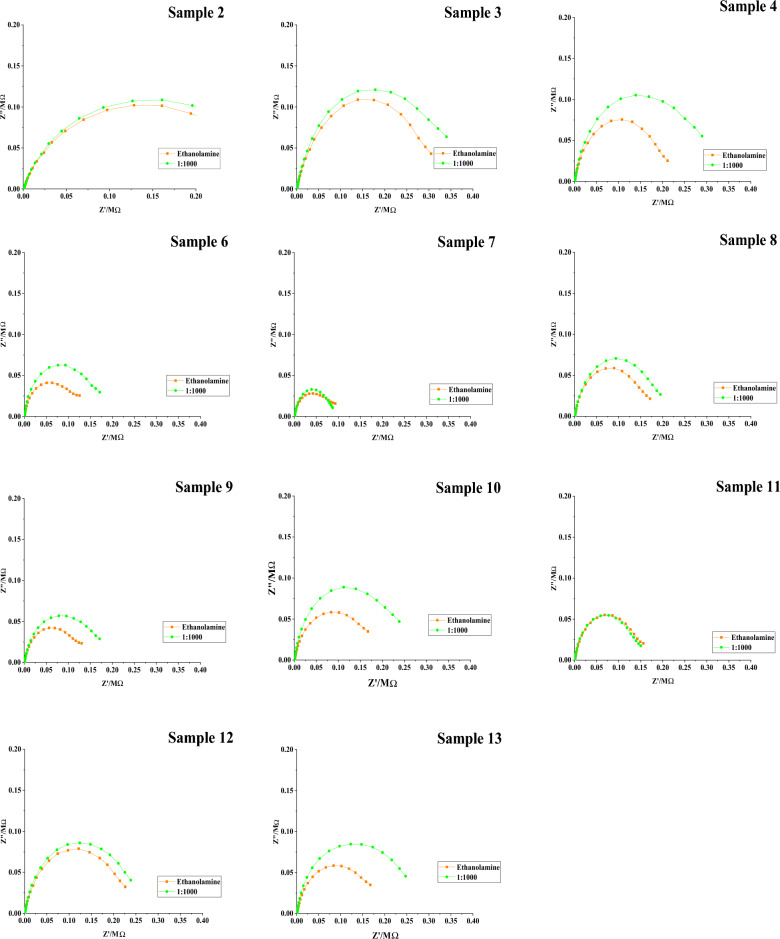
Electrochemical impedance spectroscopy for SARS-CoV-2 S antigen biosensor. 13 positive nasopharyngeal samples based on the RAPID COVID-19 antigen detection system were chosen. Samples were diluted 1:1000 and incubated for 15 min.

**Figure 8:**
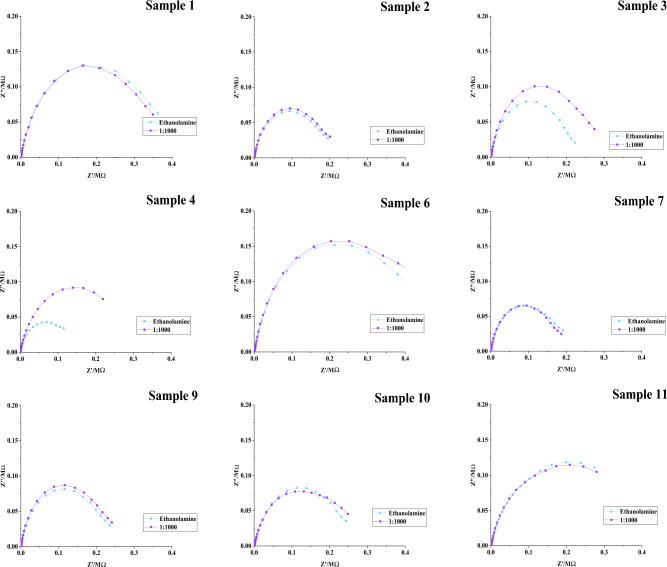
Electrochemical impedance spectroscopy for SARS-CoV-2 S antigen biosensor. 12 negative nasopharyngeal samples based on the RAPID COVID-19 antigen detection system were chosen. Sample was diluted 1:1000 and incubated for 15 min.

The anti-S biosensors were then validated using positive and negative clinical COVID-2 nasopharyngeal specimens diluted to 1:1000 for measurement of capacitance, which was then converted to ng/mL concentrations (Figs. 7 and 8, Table 1). The correlation was 84.6% for positive samples and 75% for negative samples (Table 2). However, if RAPID antigen test results were considered as reference or gold standard then the sensitivity and specificity of developed EIS would be 86% and 82%, respectively.

**Table 1:** Anti S IgY based Impedance spectroscopy results of nasopharyngeal clinical samples collected from SARS CoV-2 suspected cases.

Sample no.	Capacitance (nF)	Concentration (ng/mL)**
Positive samples*		
1	157	47,476.97
2	15	0.135
3	17	0.16
4	25	0.3325
5	36	0.8943
6	65	12.1311
7	70	19.017
8	27	0.3981
9	57	5.909
10	95	180.0313
11	0	0.0351
12	12	0.1033
13	88	95.9424
Negative samples*		
1	0	0.0351
2	2	0.042
3	42	1.533
4	72	22.763
5	− 90	0.0001
6	2	0.042
7	0	0.0351
8	− 50	0.0004
9	13	0.113
10	− 4	0.0245
11	− 5	0.022

*Classified as SARS CoV-2 positive or negative based on SARS CoV-2 RAPID assay.

**Concentrations in ng/mL were calculated based on capacitance data fitting and using the standard curve.

**Table 2:** Correlation between SARS-CoV2 S antigen detection using electro impedance spectroscopy assay and lateral flow antigen detection (RAPID Ag assay).

	Impedance spectroscopy		Sum
	Positive	Negative	
Lateral flow RAPID Ag			
Positive	11 (84.6%)	2 (15.4%)	13
Negative	3 (25%)	9 (75%)	12
Sum	14	11	25

We have included a table for fitted date from capacitance for each electrode (n = 7), before and after normalization with ethanolamine (blocking step) in the Supplementary file (Supplementary Table 1).

## Discussion

In this paper, a highly sensitive biosensor with enhanced performance was developed to rapidly diagnose a wide range of diseases, including epidemic diseases like SARS-CoV-2. The biosensor is based on an electrochemical impedimetric spectroscopy utilizing chicken IgY antibodies. The selection of the spike protein as the target analyte was due to its high immunogenicity and specific capability to detect SARS-CoV-2, as well as its lower sequence similarities to earlier SARS-CoV and MERS viruses compared to other proteins used in the literature^26,27^.

The use of egg-derived IgY polyclonal antibodies offers several advantages over antibodies produced from other sources, such as their specificity against highly conserved mammalian antigens and cost-effective and rapid extraction procedures in addition to being stressful free to animal^28–30^. The yield of purified IgY was comparable with other studies that showed a yield of 18.5–31.5 mg per egg^31,32^. However, IgY polishing using affinity purification reduced the yield but removed artifacts that may affect the biosensor fabrication and consequently its sensitivity.

Electrochemical impedance spectroscopy (EIS) was employed to measure the impedance change of the working electrode surface during the process of adding each layer of cysteamine, glutaraldehyde, and anti-spike antibody. The results were comparable to those achieved in previous studies^7,8,27,33^.

The biosensor exhibited remarkable sensitivity and linearity, enabling the detection of a broad spectrum of spike protein concentrations, ranging from 11.56 to 740 ng/mL, with a detection limit as low as 5.65 pg/mL. Cyclic voltammetry analysis provided additional confirmation of the sensor's sensitivity and linearity^34^.

The calibration curves showed that the developed electrochemical sensor outperformed previous studies in terms of sensitivity, linearity, and wider detection range^7,8,26,27,33^. This makes the sensor highly suitable for building models in portable devices for clinical applications.

Random clinical SARS-CoV-2 samples were tested on the immunosensor, confirming its ability to efficiently detect the spike protein in both recombinant and real samples. However, further investigations are needed to optimize the detection precision and incubation time. In comparison to other studies who could reach as low as 0.01 ag/mL detection limit^35^ our rapid sample analysis (15 min) is considered as good achievement that comply with POC time saving requirements.

As RT-PCR was the gold standard for any test for the calculation of sensitivity and specificity, the lack of PCR confirmed clinical samples due to pandemic cessation was a big constraint for our study. However, the reference technique in this study, the RAPID N detection system, revealed 63% to about 75% sensitivity when compared to nucleic acid based techniques^36,37^. Moreover, the fact that the lateral flow test is based on the measurements of N and not S antigen may affect decision about sensitivity and specificity.

In conclusion, the developed IgY based immunosensor shows great potential for the rapid diagnosis of various diseases, particularly epidemic outbreaks like SARS-CoV-2, making it a valuable tool for clinical applications.

### Supplementary Information

Supplementary Information.

## Data Availability

All data generated or analyzed during this study are included in this published article and its supplementary material.
