# Supplementary material for: The development of an electrochemical immunosensor utilizing chicken IgY anti-spike antibody for the detection of SARS-CoV-2
Source: Sci Rep. 2024 Jan 7;14:748. doi: 10.1038/s41598-023-50501-w (PMC10772103; doi:10.1038/s41598-023-50501-w)
Supplement: Supplementary file 1 — Supplementary Information. [file 41598_2023_50501_MOESM1_ESM.docx]

**The Development of an Electrochemical Immunosensor Utilizing Chicken IgY Anti-Spike Antibody for the Detection of Sars-CoV-2**

Khaled M. Al-Qaoud^1^, Yusra M. Obeidat^2*^, Tareq Al-Omari^1^, Mohammad Okour^1^, Mariam M. Al-Omari^3^, Mohammed IA. Ahmad^4,5^, Raed Alshadfan^4^ and AbdelMonem M. Rawashdeh^6^

1. Department of Biological Sciences, Faculty of Science, Yarmouk University, Irbid, Jordan
2. Department of Electronics Engineering, Hijjawi Faculty for Engineering Technology, Yarmouk University, Irbid, Jordan
3. Department of Basic Medical Sciences, Faculty of Medicine, Yarmouk University, Irbid, Jordan
4. Rawgene Biotech, Umm Khelad St. 33, Amman, Jordan and Atlas Medical, Sahab Industrial Area, Amman
5. Department of Chemical Sciences, Faculty of Pharmacy, University of Porto, Porto, Portugal
6. Department of Chemistry, Faculty of Science, Yarmouk University, Irbid, Jordan

^*^ Corresponding Author: E-mail: yusra.obeidat@yu.edu.jo

**
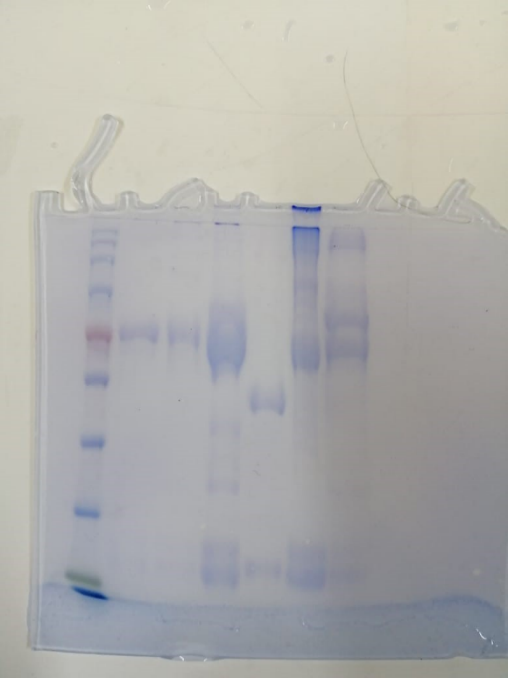

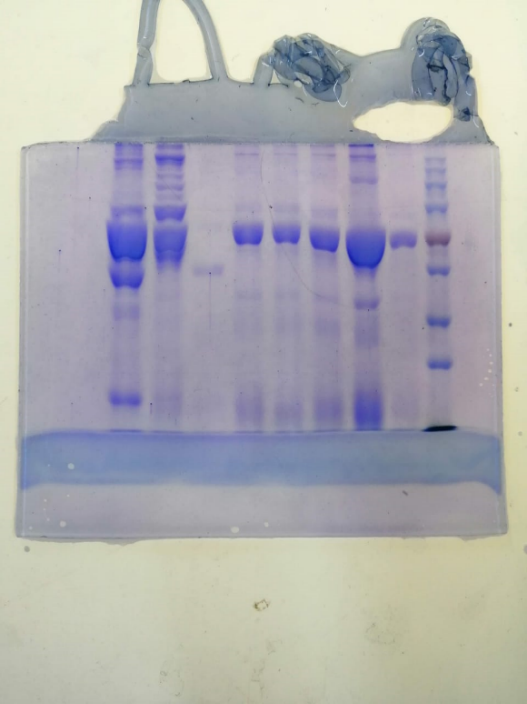
S.1 The original, unprocessed versions of the images in Figure 2 of the main manuscript**

Fig.2A Fig.2B

**S.2 The original images for the sub figures in Figure 7 of the main manuscript (including some odd samples)**

**
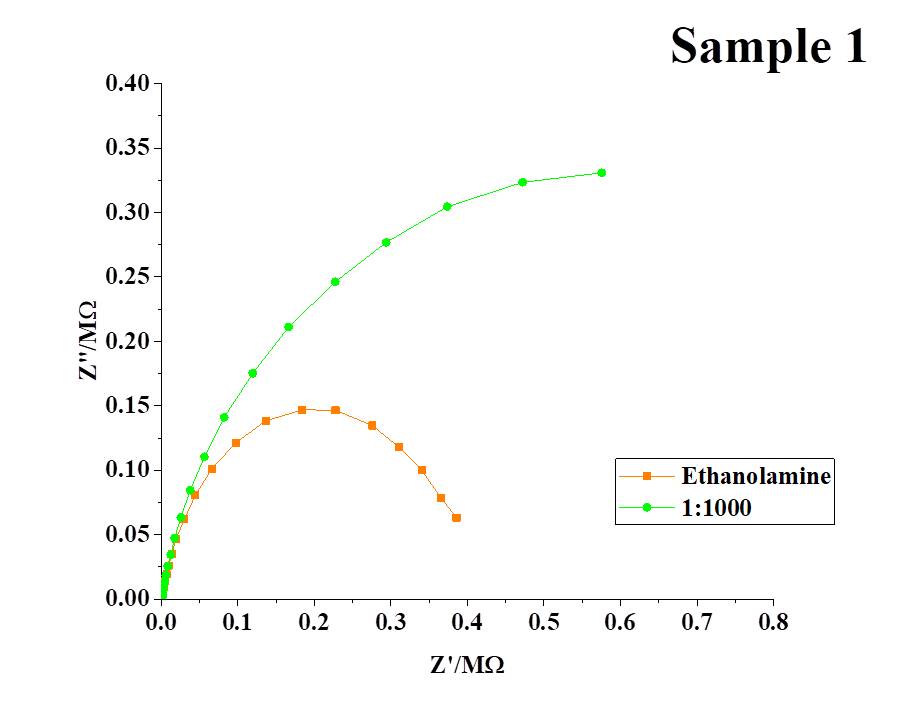
**
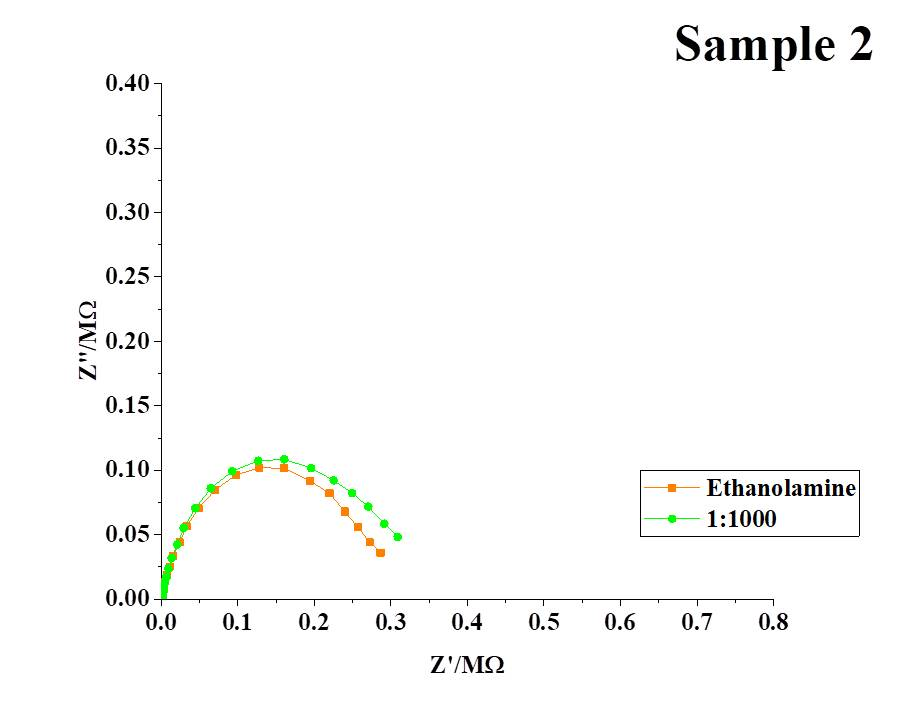


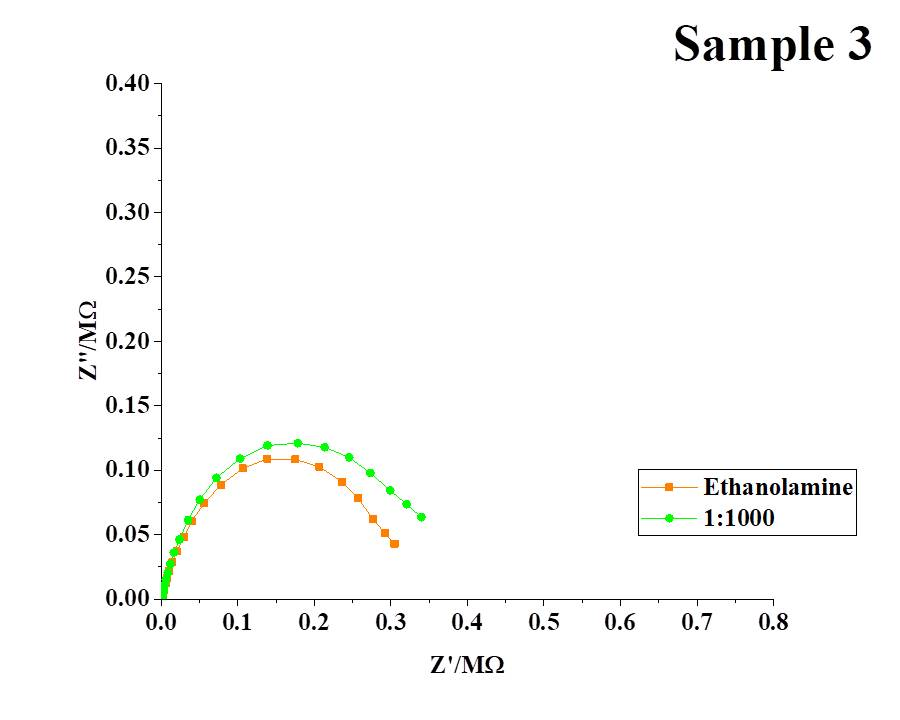

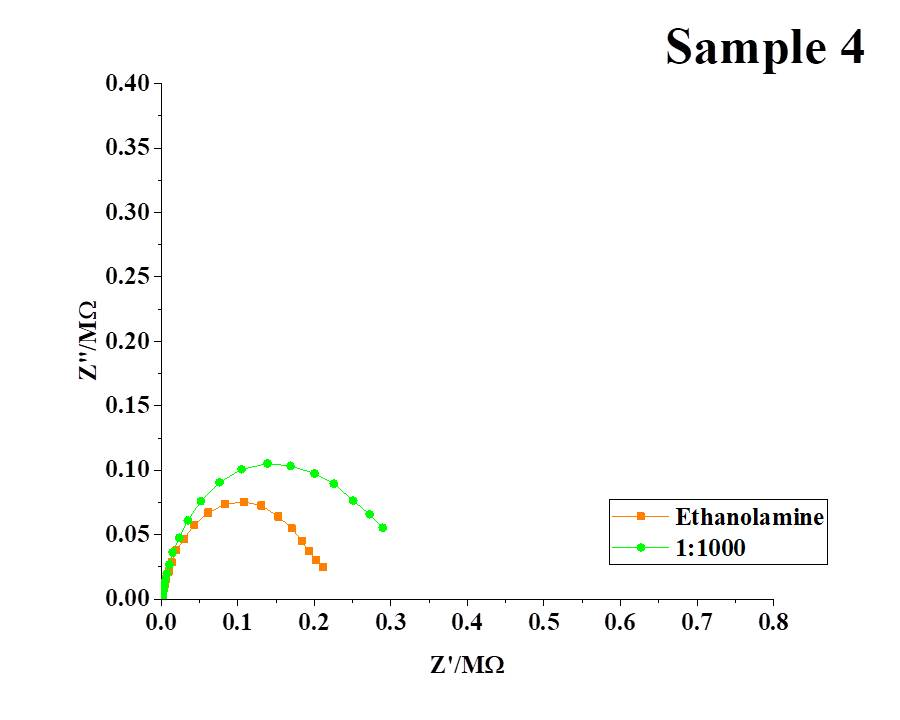


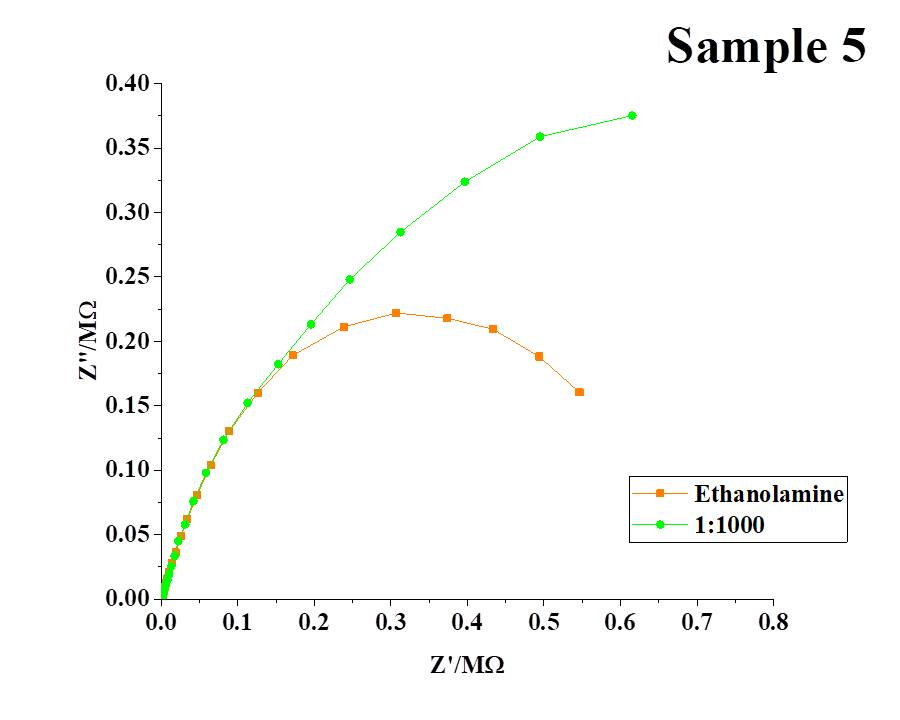

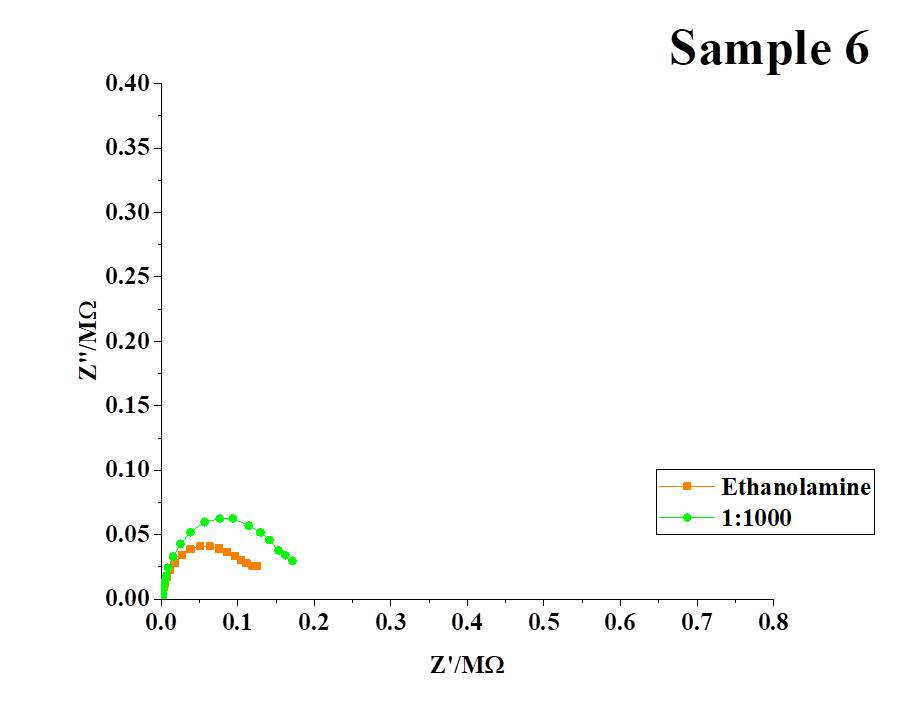


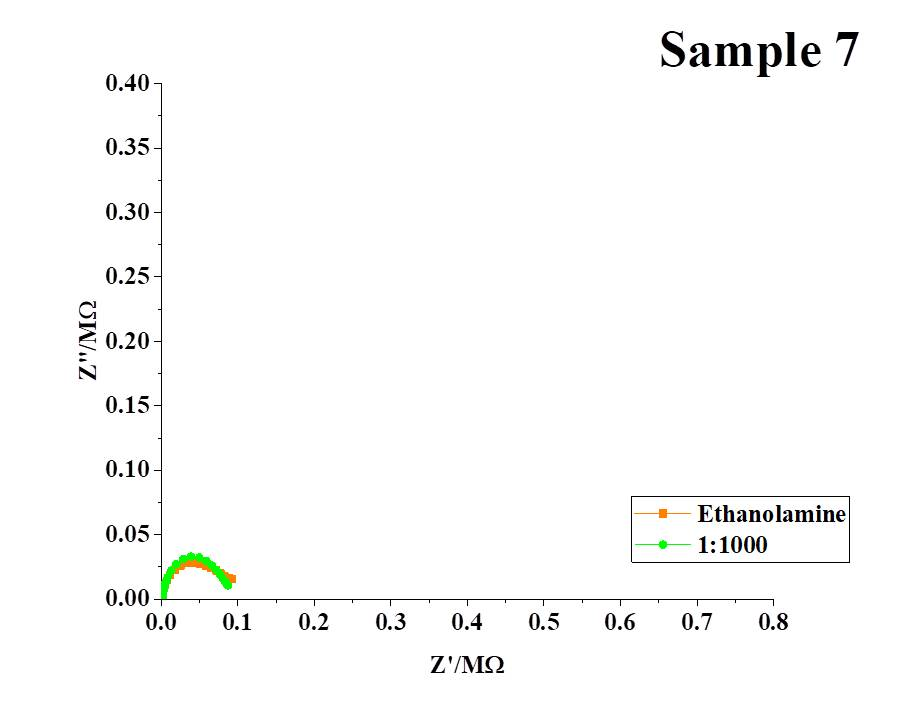

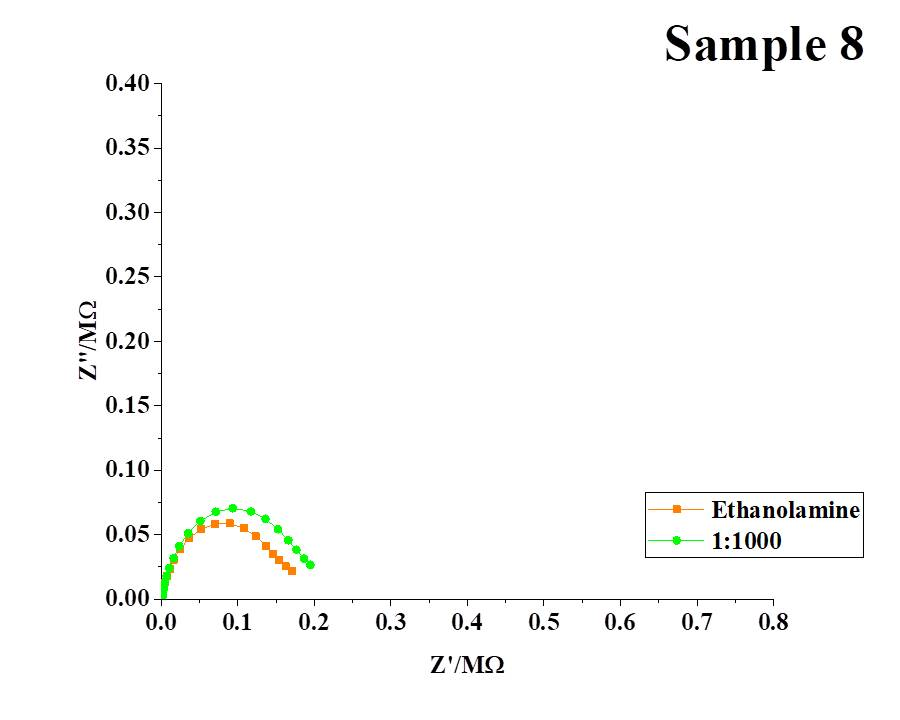


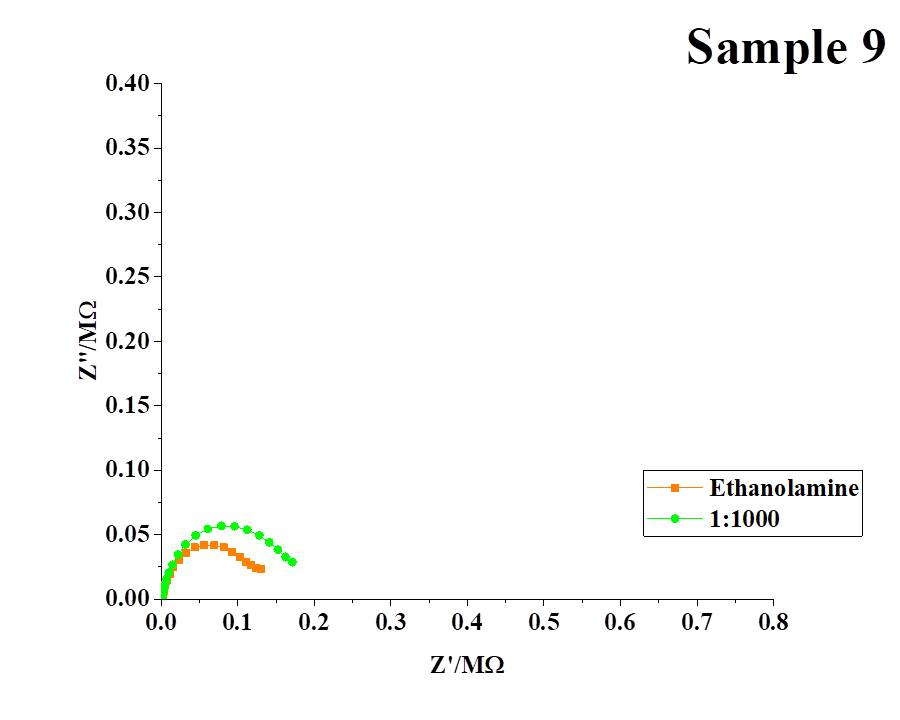

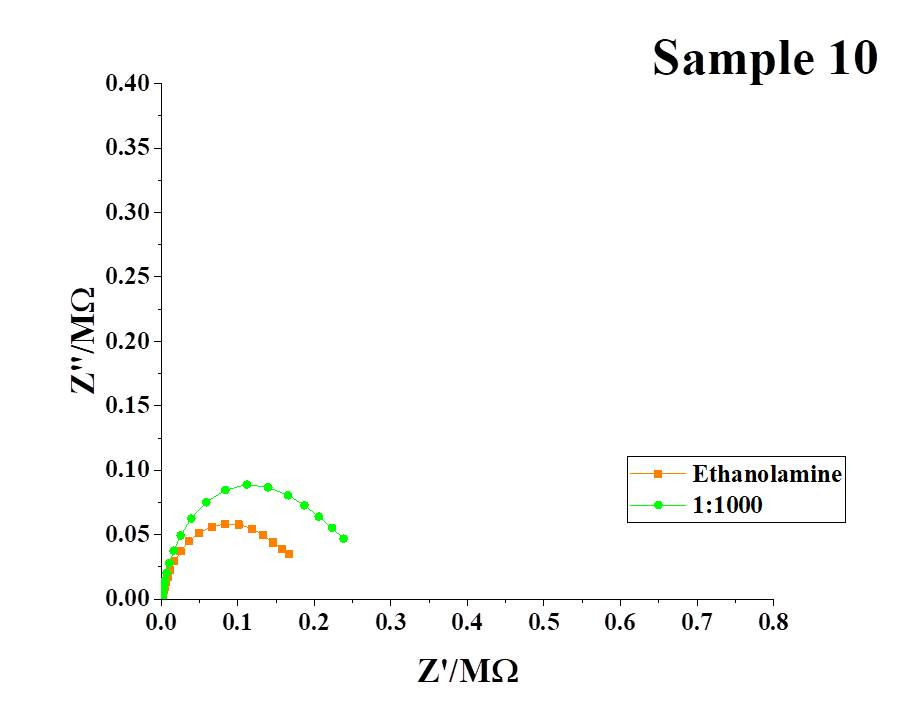


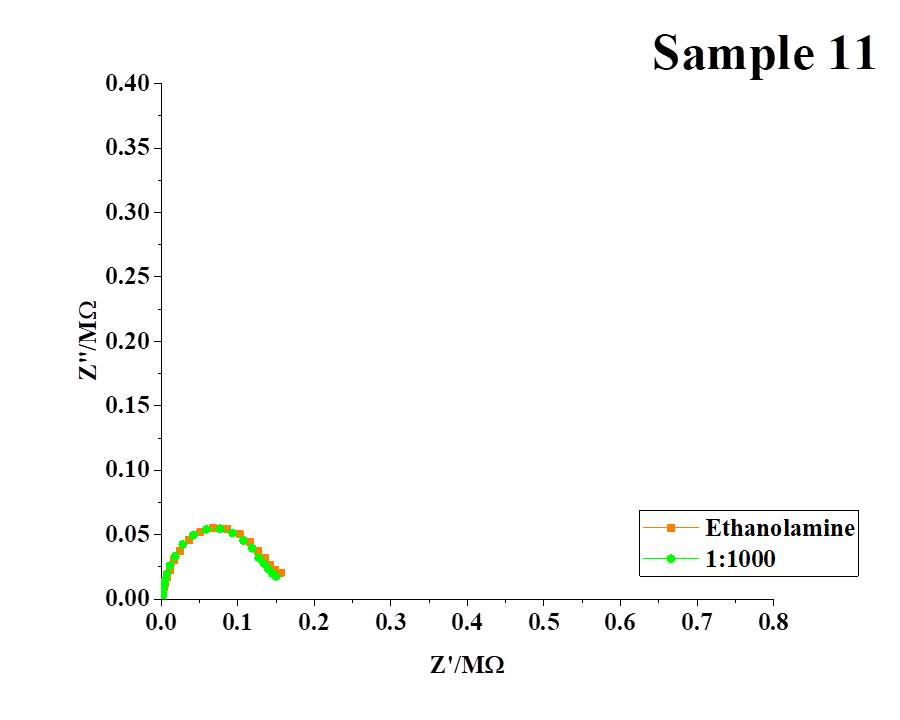

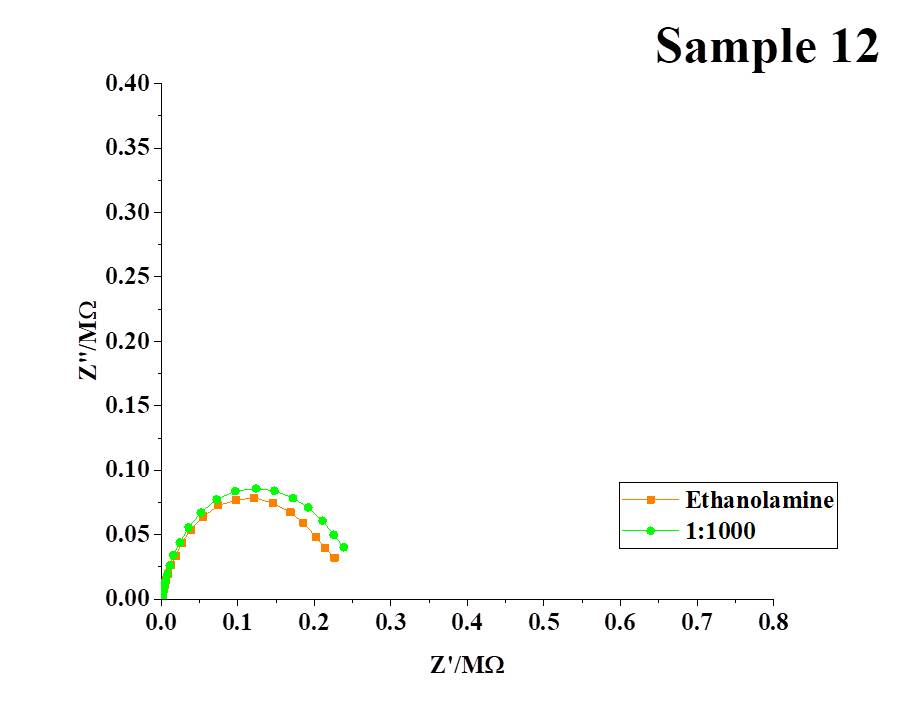


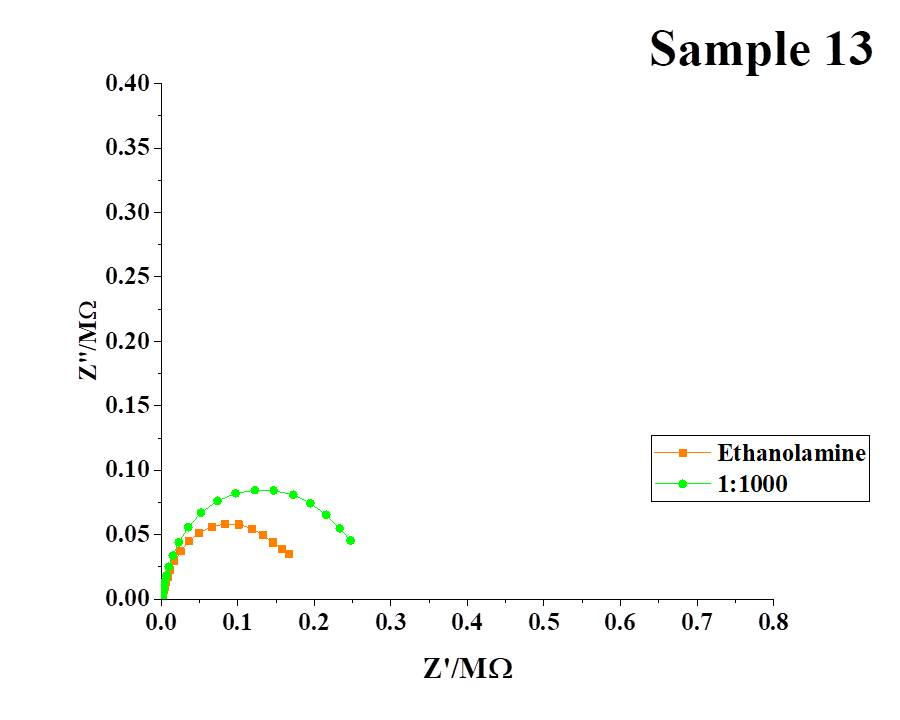


**S.3 The original images for the sub figures in Figure 8 (including some odd samples)**


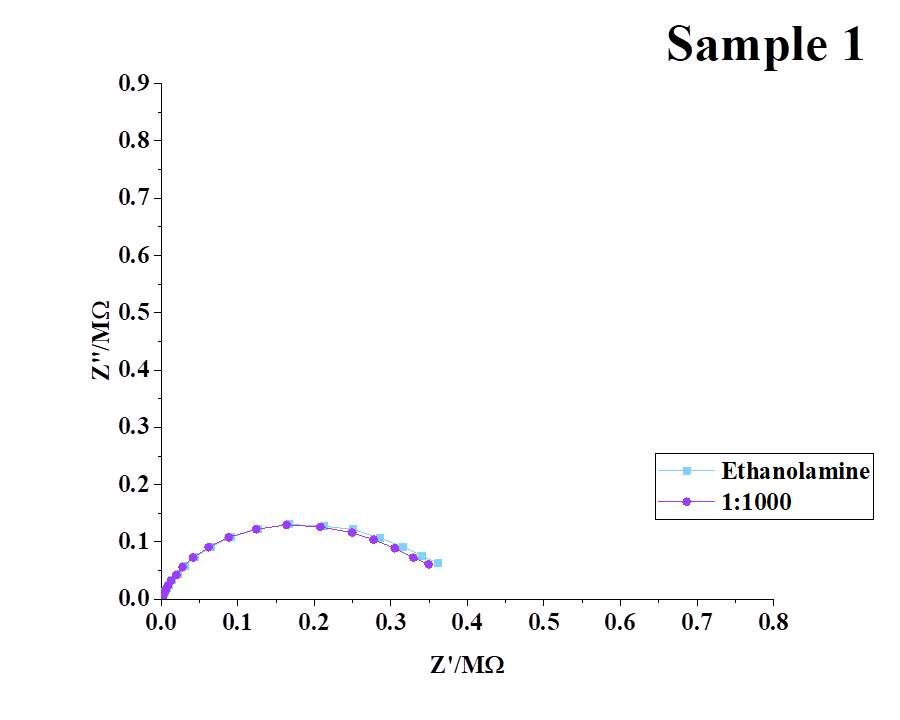

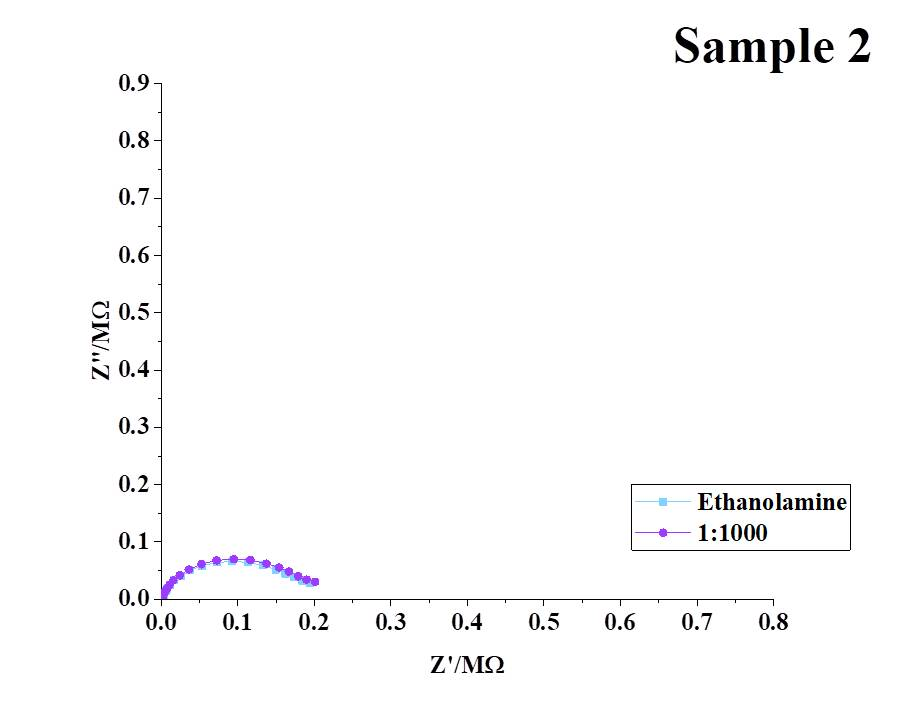


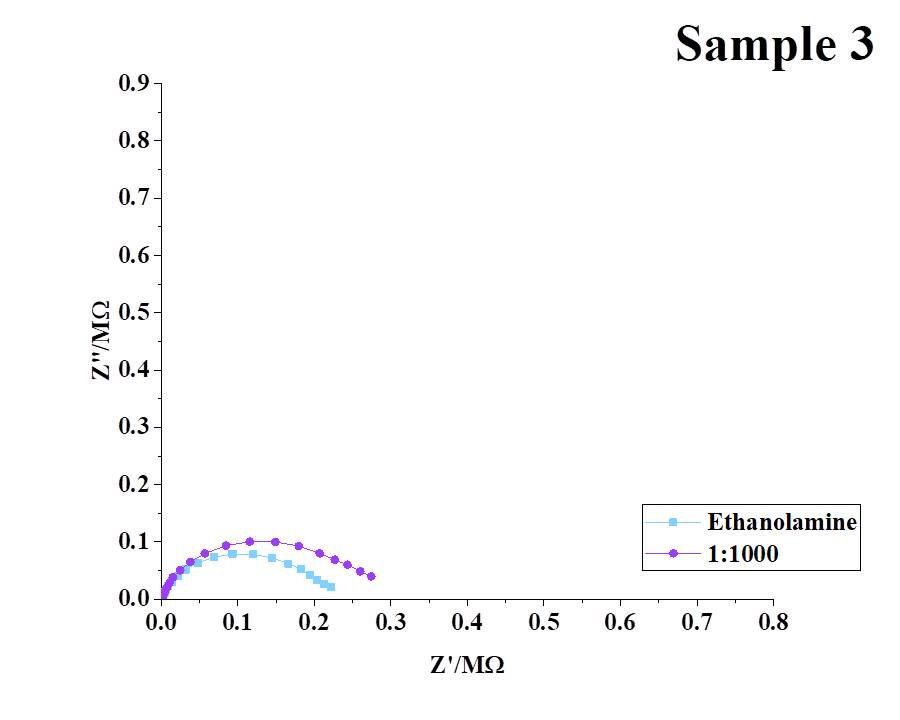

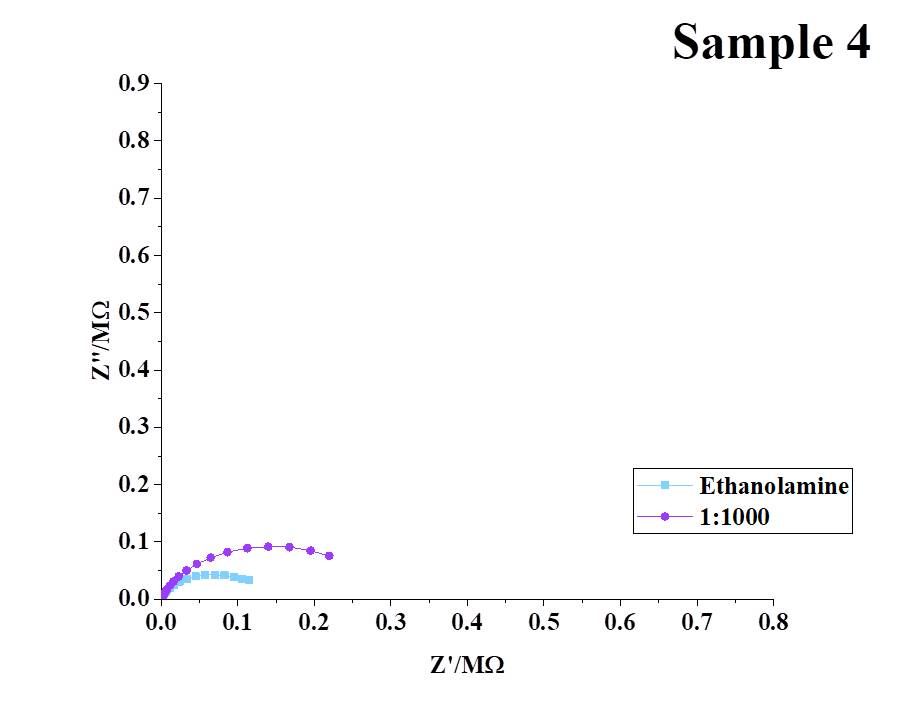


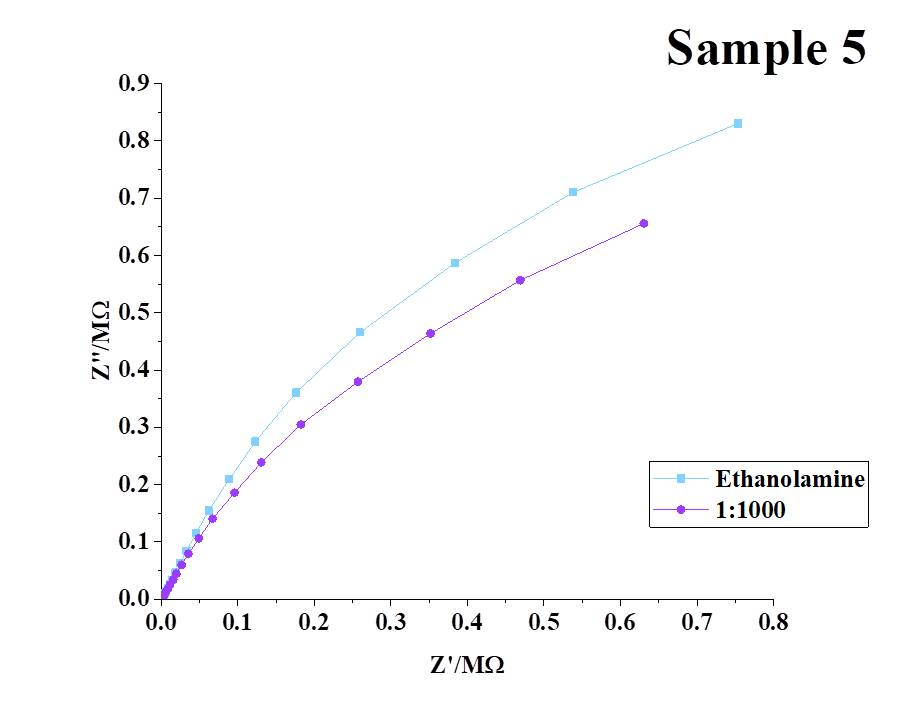

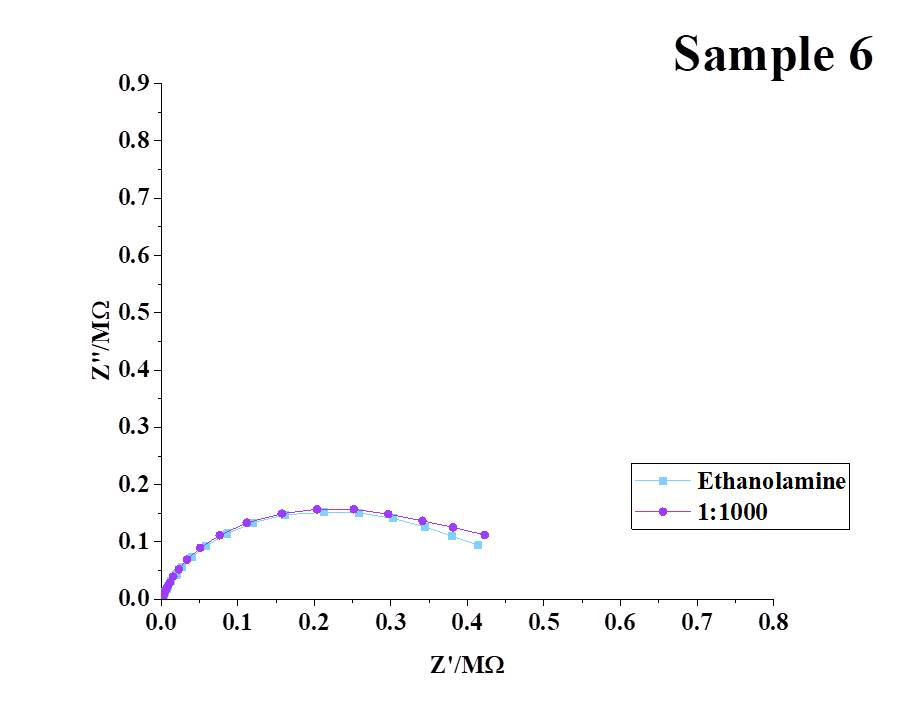


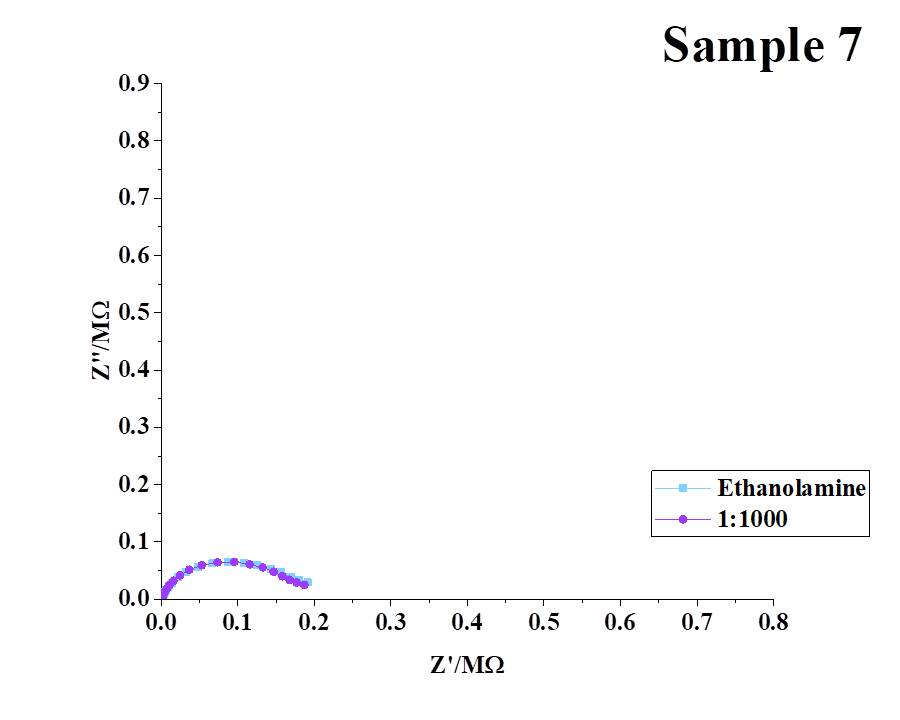

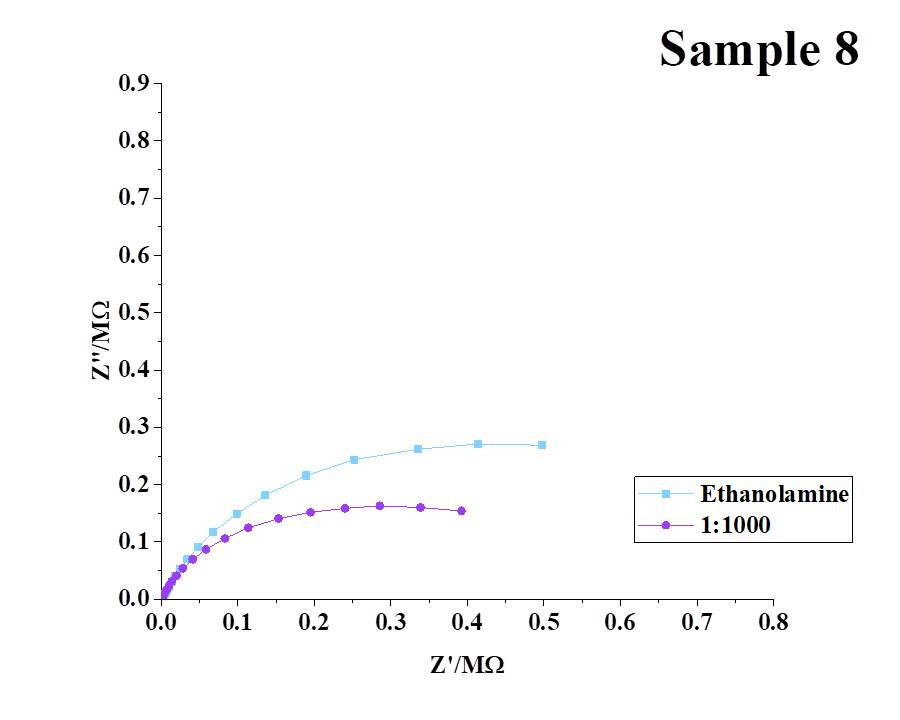


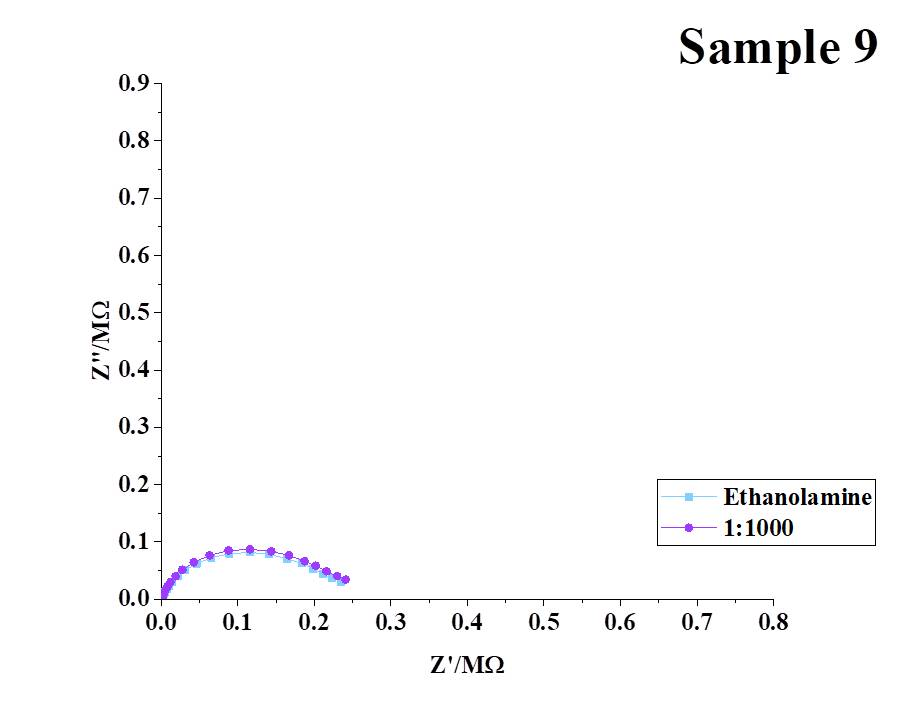

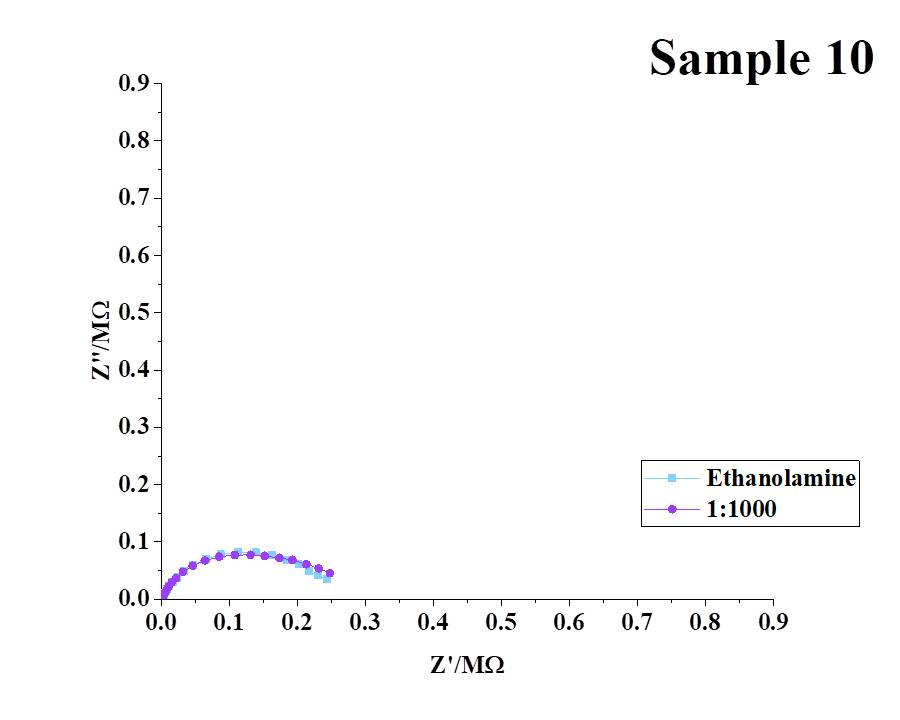


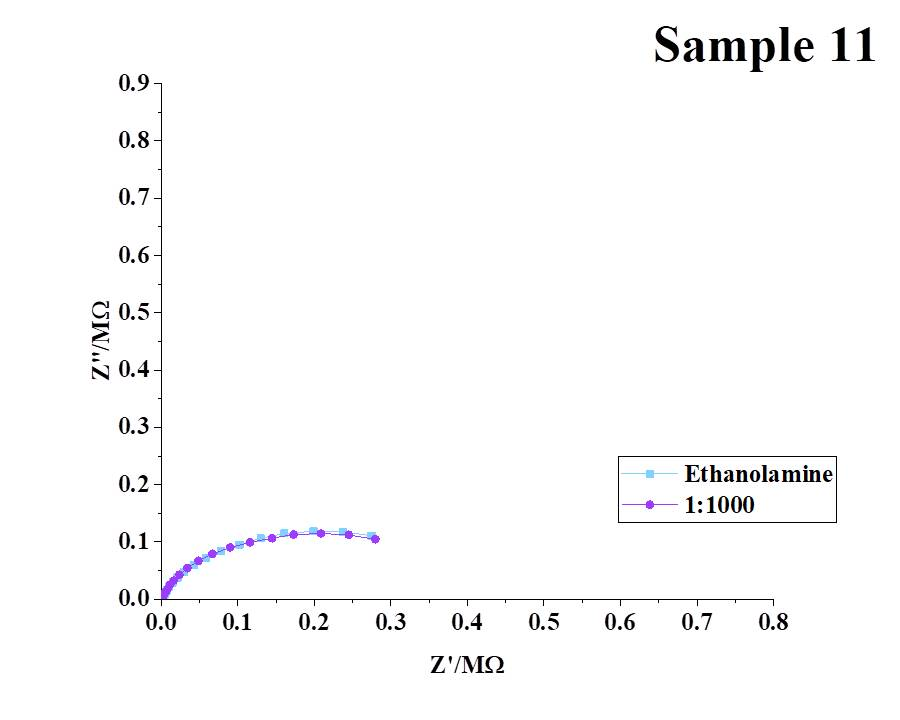


**S.4 More Electrochemical impedance spectroscopy results**


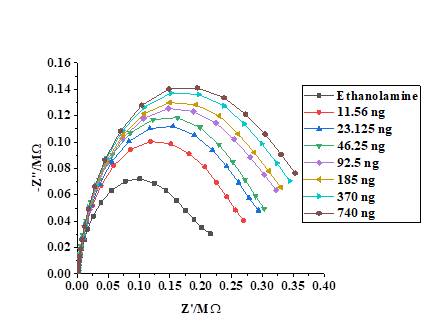


Supplementary Figure 1: R1 impedance at high concentrations (740 to 11.56 ng/ml).


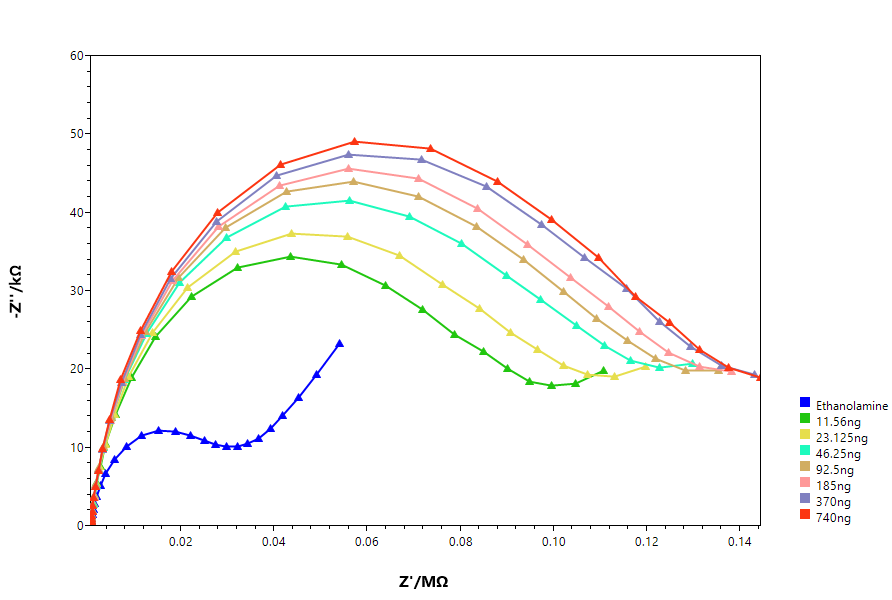


Supplementary Figure 2: R2 impedance at high concentrations (740 to 11.56 ng/ml).


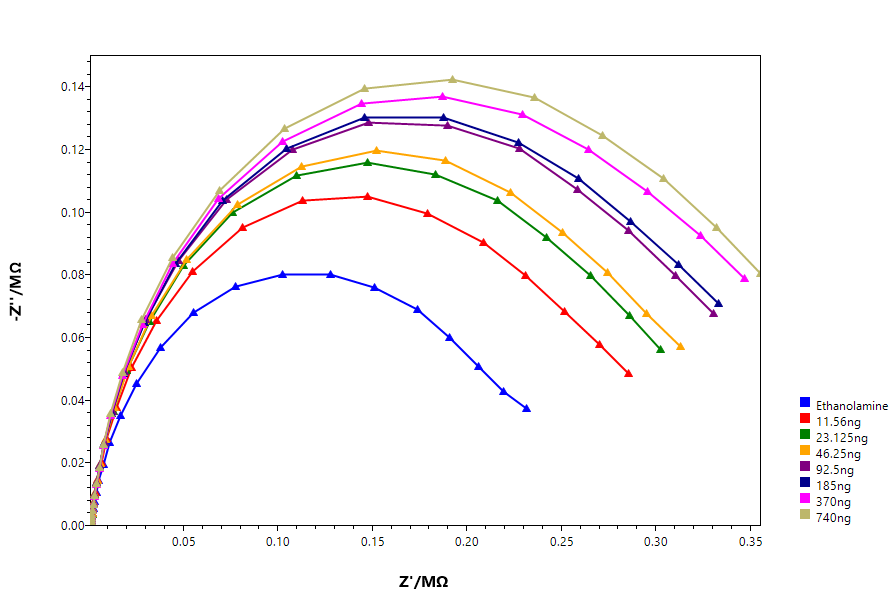


Supplementary Figure 3: R3 impedance at high concentrations (740 to 11.56 ng/ml).

Supplementary Figure 4: R4 impedance measurements for full biosensor layers with high concentrations (740 to 11.56 ng/ml).

Supplementary Figure 5: R5 impedance at lpw concentrations (11.56 ng/ml to 180 pg/ml).
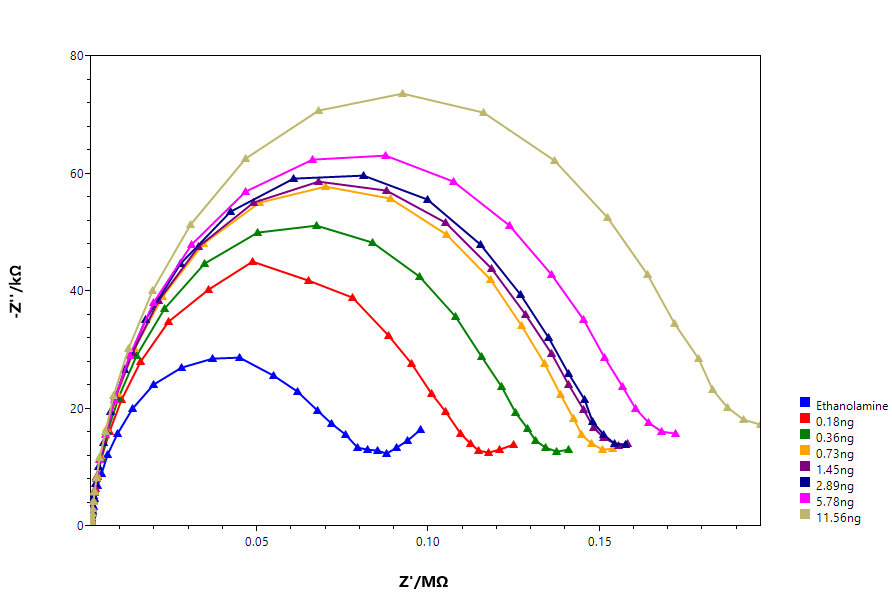


Supplementary Figure 6: R6 impedance at low concentrations (11.56 ng/ml to 180 pg/ml).

**S.5 LOD (limit of detection) results**


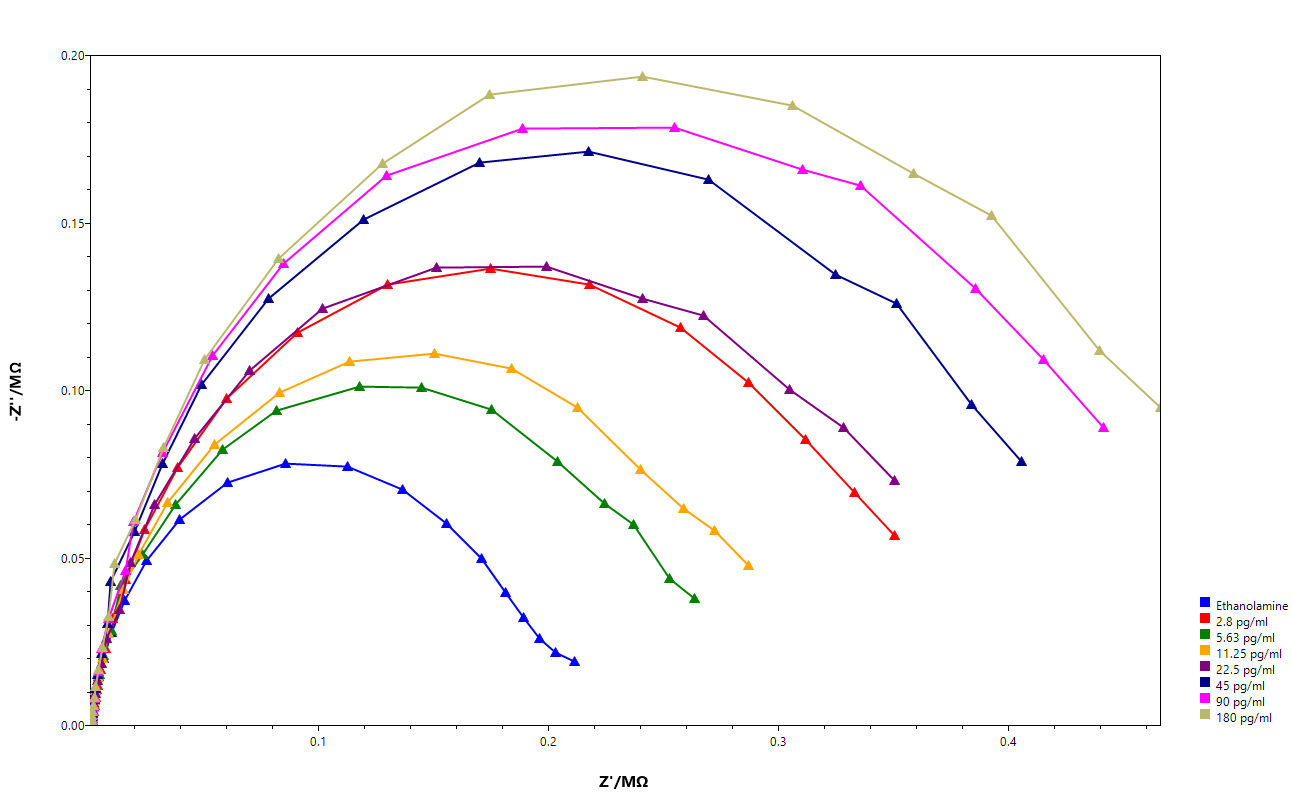


Supplementary Figure 7: R7 for limit of detection from 180 pg/ml to 2.8 pg /ml.

Supplementary Figure 8: R8 for limit of detection from 180 pg/ml to 5.65 pg /ml.

**S.6 Cyclic voltammetry results**

**
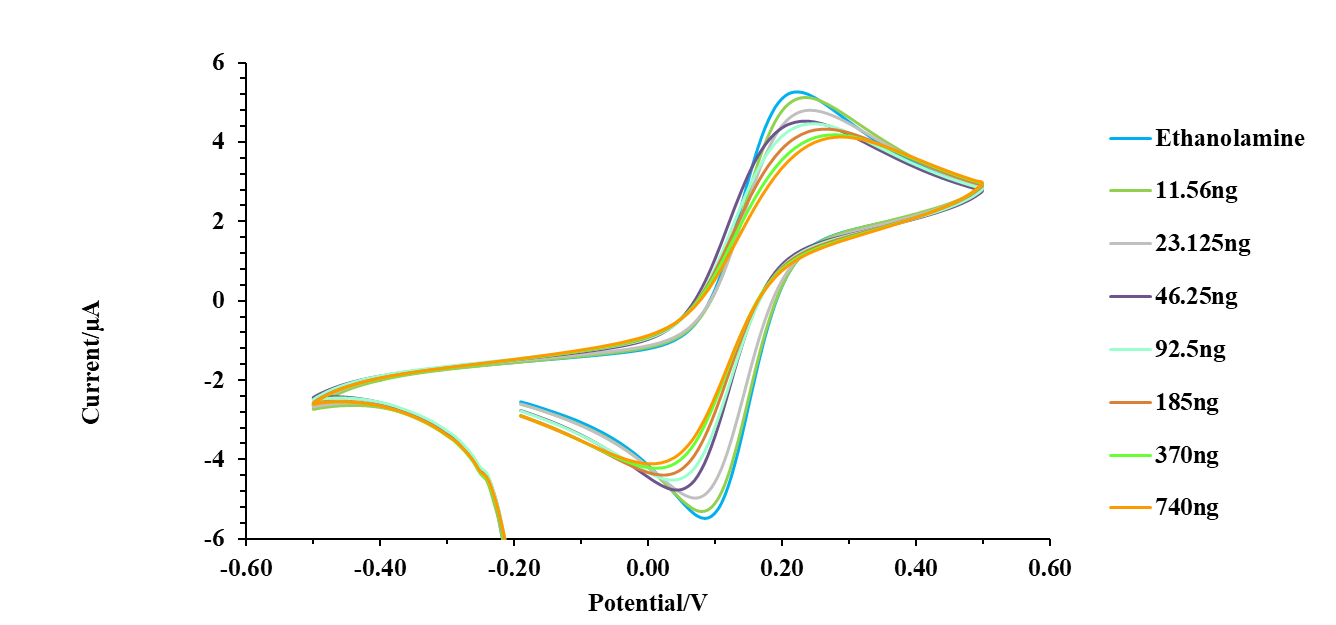
**

Supplementary Figure 9: R7 cyclic voltammetry at high concentrations (740 to 11.56 ng/ml).

**
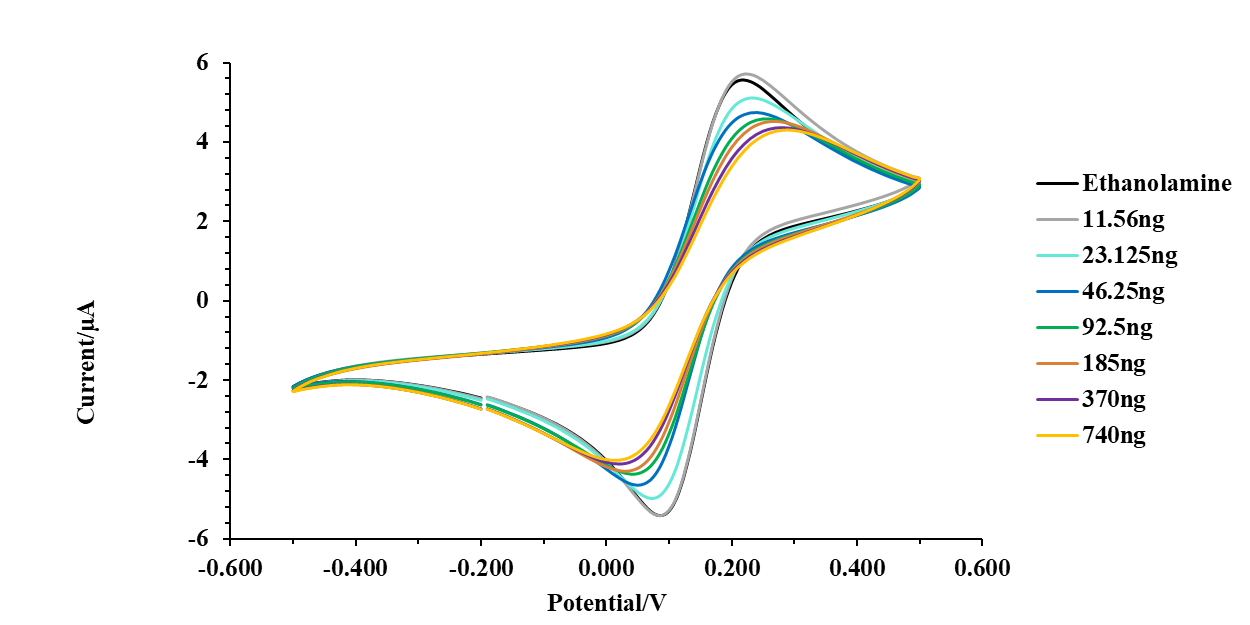
**

Supplementary Figure 10: R8 cyclic voltammetry at high concentrations (740 to 11.56 ng/ml).

**
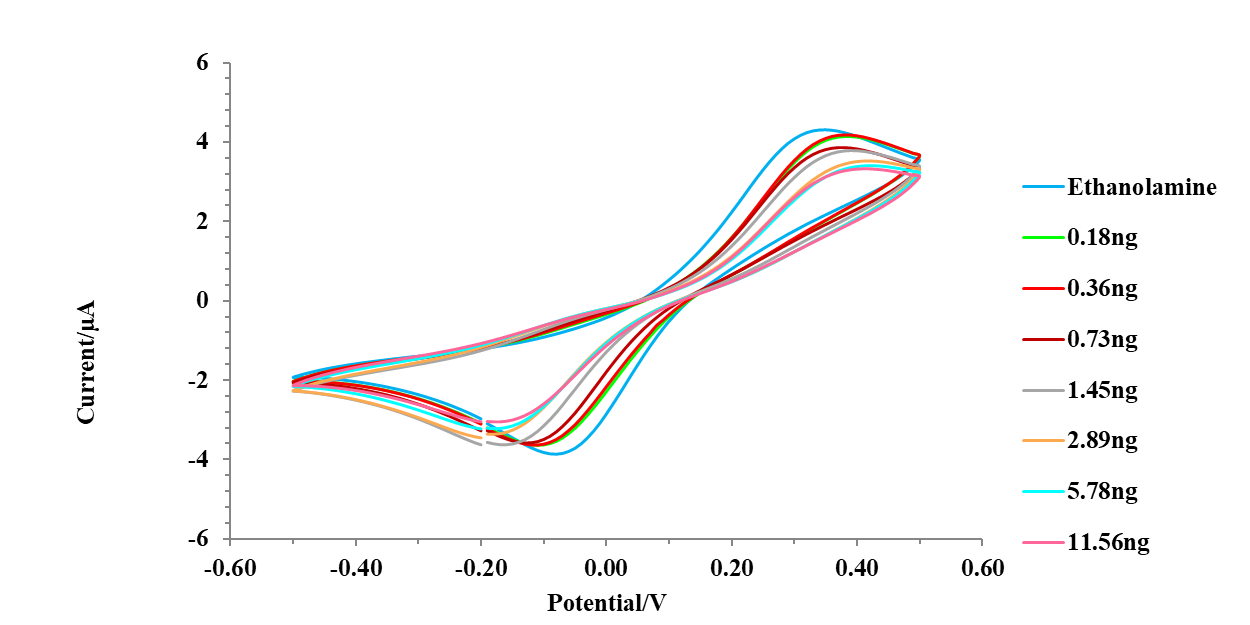
**

Supplementary Figure 11: R9 cyclic voltammetry at low concentrations (11.56 ng/ml to 180 pg/ml).

**
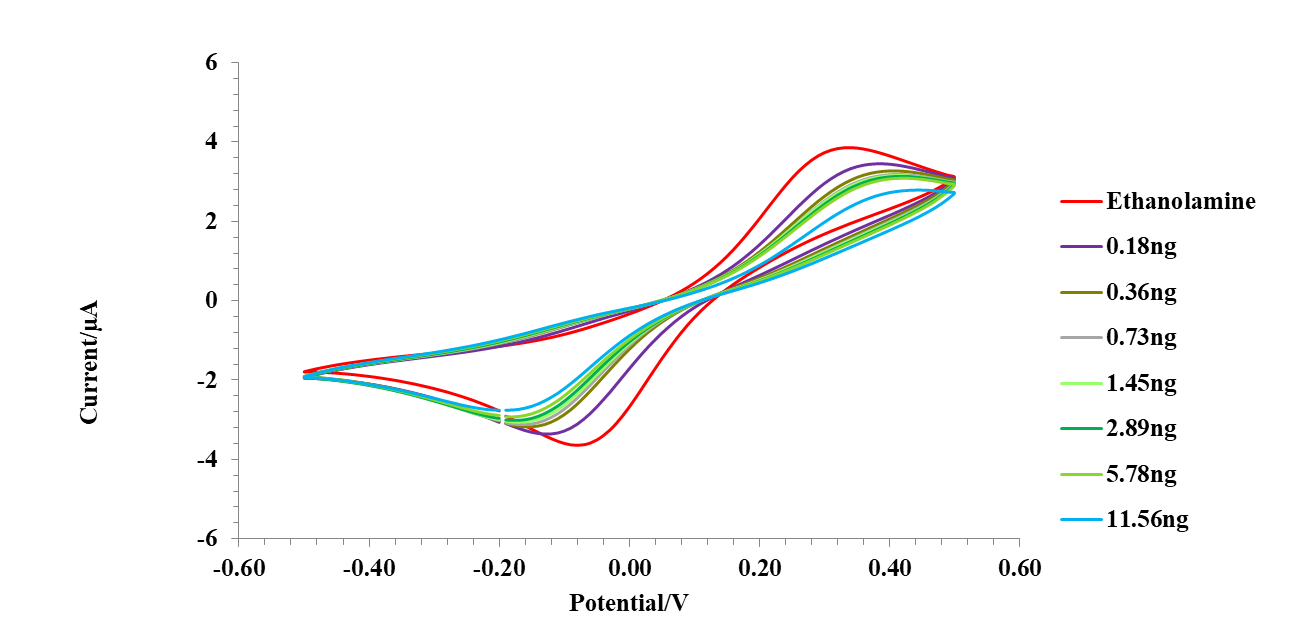
**

Supplementary Figure 12: R10 cyclic voltammetry at low concentrations (11.56 ng/ml to 180 pg/ml).

**
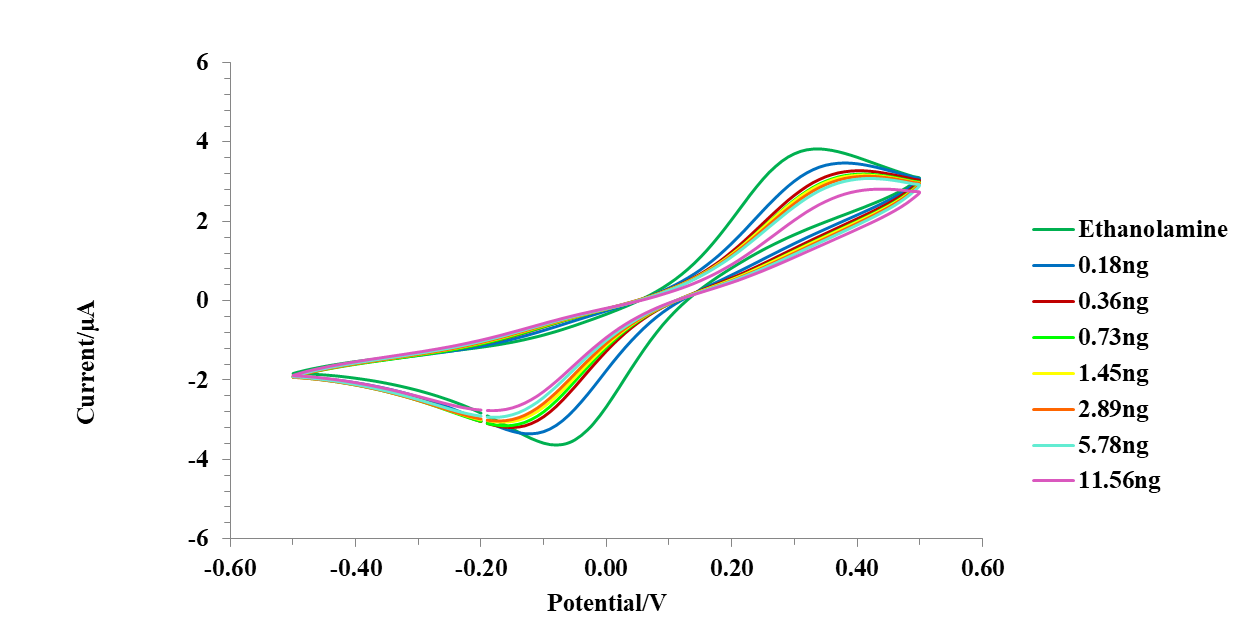
**

Supplementary Figure 13: R11 cyclic voltammetry at low concentrations (11.56 ng/ml to 180 pg/ml).

**Supplementary table 1: This table for fitted date from capacitance for each electrode (n=7), before and after normalization with ethanolamine (blocking step).**

|  | **Capacitance (before normalization with ethanolamine)** | | | | | | |
| --- | --- | --- | --- | --- | --- | --- | --- |
| **Ethanolamine** | 260 | 254 | 251 | 253 | 260 | 250 | 240 |
| **11.56ng** | 185 | 184 | 195 | 190 | 190 | 185 | 190 |
| **23.125ng** | 177 | 180 | 186 | 185 | 181 | 170 | 180 |
| **46.25ng** | 171 | 172 | 180 | 180 | 170 | 165 | 170 |
| **92.5ng** | 160 | 166 | 177 | 175 | 165 | 160 | 155 |
| **185ng** | 152 | 157 | 165 | 170 | 160 | 155 | 145 |
| **370ng** | 141 | 145 | 160 | 160 | 154 | 145 | 135 |
| **740ng** | 135 | 143 | 153 | 150 | 150 | 135 | 130 |
|  | **Capacitance (after normalization with ethanolamine)** | | | | | | |
| **Ethanolamine** | 0 | 0 | 0 | 0 | 0 | 0 | 0 |
| **11.56ng** | 75 | 70 | 56 | 63 | 70 | 65 | 50 |
| **23.125ng** | 83 | 74 | 65 | 68 | 79 | 80 | 60 |
| **46.25ng** | 89 | 82 | 71 | 73 | 90 | 85 | 70 |
| **92.5ng** | 100 | 88 | 74 | 78 | 95 | 90 | 85 |
| **185ng** | 108 | 97 | 86 | 83 | 100 | 95 | 95 |
| **370ng** | 119 | 109 | 91 | 93 | 106 | 105 | 105 |
| **740ng** | 125 | 111 | 98 | 103 | 110 | 115 | 110 |
| **x** | **y** |  |  |  |  |  |  |
|  | **Mean** | **STD** | **SEM** |  |  |  |  |
| **Concentration** | **Capacitance** | 0 | 0 |  |  |  |  |
| **11.56** | 64.14286 | 8.706866 | 3.290886 |  |  |  |  |
| **23.125** | 72.71429 | 8.596788 | 3.24928 |  |  |  |  |
| **46.25** | 80 | 8.563488 | 3.236694 |  |  |  |  |
| **92.5** | 87.14286 | 9.099974 | 3.439467 |  |  |  |  |
| **185** | 94.85714 | 8.39501 | 3.173015 |  |  |  |  |
| **370** | 104 | 9.539392 | 3.605551 |  |  |  |  |
| **740** | 110.2857 | 8.596788 | 3.24928 |  |  |  |  |
